# Supplementary material for: Statin prescription in patients with chronic obstructive pulmonary disease and risk of exacerbations: a retrospective cohort study in the Clinical Practice Research Datalink
Source: BMJ Open. 2021 Dec 7;11(12):e050757. doi: 10.1136/bmjopen-2021-050757 (PMC8655534; doi:10.1136/bmjopen-2021-050757)
Supplement: Author's manuscript [file bmjopen-2021-050757.draft_revisions.pdf]

BMJ Open is committed to open peer review. As part of this commitment we make the peer review history of every article we publish publicly available.

When an article is published we post the peer reviewers' comments and the authors' responses online. We also post the versions of the paper that were used during peer review. These are the versions that the peer review comments apply to.

The versions of the paper that follow are the versions that were submitted during the peer review process. They are not the versions of record or the final published versions. They should not be cited or distributed as the published version of this manuscript.

BMJ Open is an open access journal and the full, final, typeset and author-corrected version of record of the manuscript is available on our site with no access controls, subscription charges or pay-per-view fees (http://bmjopen.bmj.com).

If you have any questions on BMJ Open's open peer review process please email info.bmjopen@bmj.com

BMJ Open

Statin prescription in patients with chronic obstructive pulmonary disease and risk of exacerbations: a retrospective cohort study in the Clinical Practice Research Datalink

Journal:	*BMJ Open*
Manuscript ID	bmjopen-2021-050757
Article Type:	Original research
Date Submitted by the Author:	03-Mar-2021
Complete List of Authors:	Smith, Margaret; University of Oxford, Nuffield Department of Primary Care Health Sciences; Oxford University Hospitals NHS Foundation Trust, NIHR Oxford Biomedical Research Centre<br>Ashdown, Helen; University of Oxford, Nuffield Department of Primary Care Health Sciences<br>Sheppard, James; University of Oxford, Nuffield Department of Primary Care Health Sciences<br>Butler, Christopher C.; University of Oxford, Nuffield Department of Primary Health Care Sciences<br>Bankhead, Clare; University of Oxford, Nuffield Department of Primary Care Health Sciences; Oxford University Hospitals NHS Foundation Trust, NIHR Oxford Biomedical Research Centre
Keywords:	Chronic airways disease < THORACIC MEDICINE, Adult thoracic medicine < THORACIC MEDICINE, THERAPEUTICS

SCHOLARONE™
Manuscripts

I, the Submitting Author has the right to grant and does grant on behalf of all authors of the Work (as defined in the below author licence), an exclusive licence and/or a non-exclusive licence for contributions from authors who are: i) UK Crown employees; ii) where BMJ has agreed a CC-BY licence shall apply, and/or iii) in accordance with the terms applicable for US Federal Government officers or employees acting as part of their official duties; on a worldwide, perpetual, irrevocable, royalty-free basis to BMJ Publishing Group Ltd ("BMJ") its licensees and where the relevant Journal is co-owned by BMJ to the co-owners of the Journal, to publish the Work in this journal and any other BMJ products and to exploit all rights, as set out in our licence.

The Submitting Author accepts and understands that any supply made under these terms is made by BMJ to the Submitting Author unless you are acting as an employee on behalf of your employer or a postgraduate student of an affiliated institution which is paying any applicable article publishing charge ("APC") for Open Access articles. Where the Submitting Author wishes to make the Work available on an Open Access basis (and intends to pay the relevant APC), the terms of reuse of such Open Access shall be governed by a Creative Commons licence – details of these licences and which Creative Commons licence will apply to this Work are set out in our licence referred to above.

Other than as permitted in any relevant BMJ Author's Self Archiving Policies, I confirm this Work has not been accepted for publication elsewhere, is not being considered for publication elsewhere and does not duplicate material already published. I confirm all authors consent to publication of this Work and authorise the granting of this licence.

For peer review only

Statin prescription in patients with chronic obstructive pulmonary disease and risk of exacerbations: a retrospective cohort study in the Clinical Practice Research Datalink

Dr Margaret C Smith[1,2], Dr Helen F Ashdown[1], Dr James P Sheppard[1,2], Professor Chris Butler[1] , Professor Clare Bankhead[1,2]

[1] Nuffield Department of Primary Care Health Sciences, University of Oxford, Oxford, UK

[2] NIHR Oxford Biomedical Research Centre, Oxford University Hospitals NHS Foundation Trust

Corresponding Author: Dr Margaret Smith,

Nuffield Department of Primary Care Health Sciences, University of Oxford, Radcliffe Primary Care Building, Radcliffe Observatory Quarter, Woodstock Road, Oxford, OX2 6GG.

Margaret.Smith@phc.ox.ac.uk

Word count of main text 3191:

Number of Tables: 3; Number of Figures: 1

Key words: chronic obstructive pulmonary disease, exacerbation, statin, death, urinary tract infection, cohort study

For peer review only - http://bmjopen.bmj.com/site/about/guidelines.xhtml

ABSTRACT

Objective

Observational studies have suggested a beneficial effect of taking statins on frequency of chronic obstructive pulmonary disease (COPD) exacerbations. However, clinical trials of statins in people with COPD did not confirm those results. This study aimed to further investigate this association using a different methodological approach, which reduces the biases associated with previous observational study designs.

Design

Retrospective cohort study comparing new-users of statins with non-users.

Setting

General practices in England contributing to the Clinical Practice Research Datalink in 2007-2017, with linkage to data on Hospital Episode Statistics inpatient episodes.

Participants

48,124 people with COPD, aged over 40 years, who had not been prescribed statin in the previous year.

Exposure

Participants became new-users of statins at their first prescription for a statin during follow-up. They were then assumed to remain statin users. Statin users were compared with non-users.

Outcomes

Primary outcomes were COPD exacerbation, or severe exacerbation requiring hospitalisation. Secondary outcomes were death from any cause (for comparison with other studies) and urinary tract infection (negative-control). Maximum follow-up was 3 years. Adjusted hazard ratios (HR) were calculated using time-dependent Cox regression. The Andersen-Gill model was used for recurrent exacerbations. Covariates included demographic variables, variables related to COPD severity, cardiovascular comorbidities as time-dependent variables, and other comorbidities at baseline.

For peer review only

Results

7,266 participants became new-users of statins over an average 2.5 years of follow-up. In total 30,961 people developed an exacerbation, 8,110 severe exacerbation, 3,650 urinary tract infection and 5,355 died. Adjusted HR (95% CI) in statin users compared to non-users were: first exacerbation 1.01 (0.97-1.06), severe exacerbation 0.92 (0.85-1.00), number of exacerbations 1.00 (0.97-1.04), urinary tract infection 1.10 (0.98-1.24) and death 0.63 (0.57-0.70).

Conclusions

Statin use was not associated with a lower risk of COPD exacerbation. Statin medication should not be prescribed in routine clinical practice to reduce COPD exacerbation.

(300 words)

STRENGTHS AND LIMITATIONS OF THIS STUDY

- We used a large real-world cohort of over 48,000 people with COPD who were statin naïve at study entry.
- The exposure was defined as new statin prescription in follow-up and analysed as a time-dependent variable. New user designs are less susceptible to prevalent-user bias.
- This study used prescription dates for statins. We cannot be certain that the medication was taken.
- We included secondary outcomes and various levels of confounding adjustment to investigate potential sources of bias. As this was an observational study, we cannot be certain that residual confounding or other biases do not affect our results.

INTRODUCTION

Chronic obstructive pulmonary disease (COPD) is characterised by progressive and irreversible airflow limitation, together with sudden flare-ups or exacerbations. Frequent exacerbations accelerate loss of lung function and are associated with poorer quality of life and poor survival.[1] COPD is often associated with the presence of other co-morbidities, of which cardiovascular disease (CVD) is one of the most significant.[2] Therefore many people with COPD are likely to be recommended medication for primary or secondary prevention of CVD, including with statins.[3]

For peer review only - http://bmjopen.bmj.com/site/about/guidelines.xhtml

For peer review only

Increased levels of biomarkers reflecting systemic inflammation are also part of the pathology of COPD. Levels of inflammatory biomarkers increase during the acute phase of an exacerbation, and in individuals with stable COPD elevated levels of these biomarkers are associated with higher frequency of exacerbations. [4 5] Statins have been found to have anti-inflammatory effects, as well as reducing cholesterol.[6] Therefore there is a theoretical possibility that statins may have a beneficial role in COPD management over and above reducing cardiovascular risk.

Several observational studies have found an association between statin use and fewer exacerbations in COPD patients. [7 8] However randomised controlled trials have failed to confirm any such associations.[9] Several explanations for the conflict of trial results with those from observational studies have been proposed. Firstly, immortal time bias arises from misspecification of exposures so that there is a span of cohort follow-up during which, because of exposure definition, the outcome under study cannot occur.[10] Secondly, prevalent user bias occurs when risk of an outcome changes over time in a population exposed to a drug treatment.[11] Lastly, the population and treatment in the clinical trials were very precisely defined and unlikely to be typical of the general population.[12]

A study design in which all patients are statin naïve at study entry, and in which initiation of statins is included as a time dependent variable can reduce the likelihood of some of these biases. This study aimed to use such a study design to examine the association between statin prescription and incidence of COPD exacerbations in a general population of patients attending routine primary care.

METHODS

Study design

The study is a retrospective cohort study comparing new-users of statins with non-users. It was conducted within the Clinical Practice Research Datalink (CPRD) GOLD database, which is a collection of anonymised primary care records from ~700 UK practices. These data have been shown to be representative of the general UK population in terms of age, sex and ethnicity.[13] We used data from the three-quarters of CPRD English practices with linkage to the English Hospital Episode Statistics (HES), and mortality data from the UK Office for National Statistics (ONS), and patient-level social deprivation indices.

For peer review only - http://bmjopen.bmj.com/site/about/guidelines.xhtml

Patient and Public Involvement

As this is a retrospective database study there are limited opportunities for Patient and Public Involvement in study design or conduct. During the development of the protocol, we discussed experience of exacerbations with three people who had COPD themselves or had experience of caring for people with COPD.

Population and exposure

We included patients aged at least 40 years, who consulted between Jan 2007 and July 2017, and had a preceding Read code for COPD in their medical records within research-quality data. Research quality data fulfils basic CPRD-defined quality criteria at the practice and patient level. To confirm the presence of COPD, a forced expiratory volume in one second/forced vital capacity (FEV1/FVC) of <0.7 had to also be recorded. [14] To ensure that patients were actively consulting and that there were enough data to identify baseline values of covariates, the index date was defined as the latest of the consultation date and the date of accumulating two years of research quality data in the current practice. Patients in the selected cohort were also all statin naïve as they were only included in the cohort if there had been no statin prescription in the 12 months before the index date. They were defined as newly prescribed users of statins once a prescription was issued on the index date or during follow-up.

Outcomes

Patients were followed-up until they were censored after 3-years, or until the end of the study period or they died or left their practice or their practice stopped contributing data to CPRD GOLD, if any of these occurred within 3-years. Follow-up was limited to 3 years because we expected adherence to statins to drop-off over time.[15]

Our primary outcomes were any COPD exacerbation and severe exacerbation requiring hospitalisation. An acute exacerbation of COPD in the Primary Care record was defined as a code for acute exacerbation, code for lower respiratory tract infection, prescriptions for 5-14 days of exacerbation-specific antibiotics and oral steroid on the same date, or a symptom of exacerbation together with a prescription for exacerbation-specific antibiotics or oral steroids.[16] Exacerbations were also identified from the HES inpatient record, from acute COPD or acute respiratory infection as the primary diagnosis or from a code for acute exacerbation of COPD recorded anywhere in the hospitalisation record. We identified all exacerbations in follow-up. To prevent double counting,

For peer review only - http://bmjopen.bmj.com/site/about/guidelines.xhtml

For peer review only

events indicating exacerbations within 14 days of each other were considered to be the same exacerbation. A severe exacerbation was any exacerbation with hospitalisation recorded in the HES inpatient data.[17]

Secondary outcomes were death from any cause, and urinary tract infection (UTI). Death was chosen so that we could compare another observed association with results from randomised controlled trials in the general population. UTI recorded in Primary Care data was included as a negative-control outcome because we did not expect any association with statin use.

Covariates

We assessed potential confounders at the index date and time dependently. For the purpose of adjustment in regression analyses covariates were combined into groups: lifestyle and demographic factors, markers of COPD severity, CVD-related variables, and other co-morbidities or health-related variables.

- Lifestyle and demographic variables included gender, age at index, deprivation (as quintile of Index of Multiple Deprivation), and smoking status before index.
- Covariates related to COPD severity were FEV_1 percent of predicted, number of COPD exacerbations, severe exacerbation requiring hospitalisation, regular prescription of COPD-related medication (short-acting bronchodilator, long-acting bronchodilator, inhaled corticosteroid, nebuliser), and receiving oxygen therapy.[18 19] The most recent FEV_1 percent of predicted in the two years prior to index was extracted. All other variables were assessed in the one-year before index. COPD exacerbations were defined as for outcomes. Regular prescription was defined as at least three prescriptions in the year.
- CVD-related variables were previous CVD, diabetes and hypertension. These variables were assessed as if they had occurred ever before index and time-dependently during follow-up. We included this group of variables as time-dependent covariates in main analyses because they are very likely to be associated with statin exposure. CVD was defined as a diagnosis code for one of coronary heart disease, stroke, heart failure or peripheral artery disease in the Primary Care data or in the linked HES inpatient data. Hypertension was defined as a diagnosis code or a prescription for an antihypertensive medication. We also included body mass index (BMI) (the most recent measurement in two years before index) in this group of covariates. We also did a post hoc sensitivity analysis in which we included CVD-related variables assessed ever before index but not time dependently in follow-up.
- Comorbidities and other health-related variables including asthma, other respiratory disease (bronchiectasis, cystic fibrosis, alpha-1 anti-trypsin-deficiency, interstitial lung disease), lung

For peer review only - http://bmjopen.bmj.com/site/about/guidelines.xhtml

cancer, rheumatoid arthritis, gastro-oesophageal reflux disease and osteoporosis were assessed from codes in Primary Care and HES inpatient data recorded at any time before the index date. Depression, influenza vaccination, and number of doctor or nurse consultations were assessed in Primary Care data in the year before index.

Statistical Analysis

We used Cox regression to investigate the association of statin use with time to first event for exacerbation, severe exacerbation, death or UTI. For analyses of recurrent exacerbation we used the Andersen and Gill model.[20] Statin use was defined as a binary time-dependent variable. Patients became new-users of statins at their first prescription of statin on the index date or during follow-up. In main analyses, we took an intention to treat approach in which people were assumed to remain statin users from the initial prescription until they were censored after a maximum of 3-years of follow-up. The analyses were repeated with combinations of the different sets of covariates to try to understand the role of confounding. All analyses were conducted in Stata 16 (StataCorp, Texas, US).

Age at index and BMI were included in analyses as continuous covariates. FEV_1 percent of predicted was included as a categorical variable (≥80%, 50-<80%, 30-<50%, <30%). Quintile of IMD, number of exacerbations (0, 1, 2, 3, 4, ≥5) and smoking status (never, ex, current) were also included as categorical variables. Other covariates were included as binary variables. Time dependent variables were incorporated by splitting patient records at the date of the first prescription for statin or when CVD, diabetes or hypertension were first recorded.

Missing values of BMI or FEV_1 percent of predicted were imputed with multiple imputation by chained equations, using a linear regression model for imputing FEV_1 percent of predicted and predictive mean matching for BMI, and including all variables to be included in planned analyses (including outcomes). Thirty imputed data sets were calculated and estimates from regressions were pooled using Rubin's rules. People with missing smoking data were assumed non-smokers.

In secondary analyses, we censored 90-days after the last recorded statin prescription to investigate the effect of non-adherence on associations. A *post hoc* sensitivity analysis was conducted for the death outcome in which we adjusted for CVD variables assessed at baseline rather than the time-dependent CVD variables.

For peer review only - http://bmjopen.bmj.com/site/about/guidelines.xhtml

RESULTS

Baseline characteristics

A total of 8,375,283 people were available with complete linkage within the CPRD GOLD database (Figure 1). Within these, we identified 76,702 people aged 40+ years who fulfilled the definition of a COPD diagnosis, had a recent record of consulting their GP and had data in the study period. Of these, 48,124 were statin naïve and were included in the cohort. Of these, 7,266 had at least one statin prescription on the index date or subsequently during a maximum of 3-years of follow-up. For 40,858 there was no record of a statin prescription in follow-up.

People who subsequently started on statins had different baseline characteristics compared to those who did not (Table 1). They were less likely to be female (42% compared to 50%). They were also more likely to have social deprivation above the median (57% vs. 54%) or to be current smokers (8% vs. 10%). Variables related to COPD severity indicated that COPD was on average slightly less severe at baseline in the new statin users. Those who became statin users had a slightly higher body mass index at baseline (27.4 vs 25.9 kgm^{-2}) and considerably higher prevalence of hypertension (50% vs 39%), CVD (14% vs 8%) and diabetes (6% vs 2%) at baseline. More had hypertension, CVD or diabetes newly recorded in follow-up. New statin-users were also more like to have an influenza vaccination in the year before baseline (72% vs 66%), and more likely to make 5+ visits to a GP (57% vs 55%) or nurse (24% vs 22%).

For peer review only

Table 1. Summary statistics by category of statin user. Non-users did not have a statin prescription in follow-up. New users had at least one statin prescription on or after the index date. Statistics are n (%) unless otherwise stated.

Variable	Non-user (N=40858)	New user (N=7266)
Index date (median)	March 2008	July 2007
**Lifestyle and demographic**		
Female	20375 (50%)	3045 (42%)
Mean(SD) age (years)	67(12)	68(10)
Deprivation above median (IMD 2007)	22109 (54%)	4174 (57%)
Smoking status		
Current	4153 (10%)	604 (8%)
Ex	19978 (49%)	3661 (50%)
Never	16524 (40%)	2973 (41%)
Missing	203 (0%)	28 (0%)

For peer review only - http://bmjopen.bmj.com/site/about/guidelines.xhtml

**Related to COPD severity**		
Mean(SD) FEV_1 (percent of predicted)	63(21)	65(21)
Missing	11278 (28%)	2062 (28%)
FEV_1 percent of predicted		
≥ 80	6073 (15%)	1123 (15%)
≥ 50 - < 80	15152 (37%)	2788 (38%)
≥ 30 - < 50	6966 (17%)	1100 (15%)
< 30	1389 (3%)	193 (3%)
Missing	11278 (28%)	2062 (28%)
Number of COPD exacerbations in 1-year before index		
0	20336 (50%)	3785 (52%)
1	11154 (27%)	1914 (26%)
2	5073 (12%)	884 (12%)
3	2237 (5%)	346 (5%)
4	1024 (3%)	175 (2%)
≥5	1034 (3%)	162 (2%)
At least one severe exacerbation in 1-year before index	2981 (7%)	499 (7%)
Regular prescription (3+) of COPD medication in 1-year before index		
Short acting bronchodilator	20936 (51%)	3661 (50%)
Long acting bronchodilator	18068 (44%)	3075 (42%)
Inhaled corticosteroid	19136 (47%)	3323 (46%)
Nebuliser	1654 (4%)	230 (3%)
On oxygen therapy	423 (1%)	49 (1%)
**Related to cardiovascular disease**		
Diabetes		
Recorded before index date	1007 (2%)	462 (6%)
New over follow-up	482 (1%)	693 (10%)
Hypertension		
Recorded before index date	16093 (39%)	3661 (50%)
New over follow-up	4632 (11%)	2066 (28%)
Cardiovascular disease		
Recorded before index date	3241 (8%)	991 (14%)
New over follow-up	1812 (4%)	1984 (27%)
Body mass index (kg m^{-2}) Mean(SD)	26(6)	27(6)
Missing	10838 (27%)	1679 (23%)
**Comorbidities and other health-related recorded before index date**		
Respiratory comorbidities		

For peer review only - http://bmjopen.bmj.com/site/about/guidelines.xhtml

Asthma	10818 (26%)	1890 (26%)
Other respiratory disease	1289 (3%)	195 (3%)
Other comorbidities		
Lung cancer	331 (1%)	46 (1%)
Depression in 1-year before index	2808 (7%)	474 (7%)
Rheumatoid arthritis	700 (2%)	123 (2%)
Gastro-oesophageal reflux disease	4966 (12%)	995 (14%)
Osteoporosis	2113 (5%)	300 (4%)
Influenza vaccination in 1-year before index	27143 (66%)	5211 (72%)
Regular (5+) contacts with practice staff in 1-year before index		
GP	22525 (55%)	4153 (57%)
Nurse	8979 (22%)	1772 (24%)

SD, standard deviation; IMD, index of multiple deprivation, COPD chronic obstructive pulmonary disease; FEV_1, forced expiratory volume in one second percent of predicted; GP, general practitioner

Associations of statin use with outcomes

The mean follow-up was 2.5 years. 17,350 (36%) people were censored before the end of the maximum 3 years of follow-up, due to death (5,355 (11%)), end of the study period (1,973 (4%)), or leaving their current GP practice or the end of downloaded data in CPRD from their practice (10,022 (21%)). Over follow-up, there were a total of 106,961 exacerbations recorded. 30,961 (64%) people had at least one exacerbation, 8,110 (17%) were hospitalised for a severe exacerbation and 3,650 (8%) had at least one UTI diagnosed.

The mean follow-up after starting to use statins was 1.6 years. The first statin prescription was simvastatin in 6,089 (84%) of the new users. It was atorvastatin for 989 (14%), pravastatin for 133 (2%), rosuvastatin for 53 (1%) and fluvastatin for two people. The number of statin prescriptions per person was 9 (4-18) (median and interquartile range).

COPD exacerbation

There was no association between statins and COPD exacerbation after adjustment for basic demographic variables (hazard ratio (HR) 0.99, 95%CI 0.94-1.03) (Table 2). Additionally adjusting for cardiovascular risk factors resulted in a small statistically significant beneficial effect of statins (HR 0.94, 95%CI 0.90-0.99) (Table 2). However after further adjustment for variables related to severity of COPD there was no evidence of an association (HR 1.01, 95%CI 0.96-1.06). This remained the case after adjustment for all covariates (HR 1.01, 95%CI 0.96-1.06) and in analyses censoring 90-days after the last statin prescription (Table 3). A similar pattern of HR with different adjustments was seen for

For peer review only - http://bmjopen.bmj.com/site/about/guidelines.xhtml

For peer review only

the recurrent COPD exacerbation outcome and for severe COPD exacerbations. The HR for recurrent COPD exacerbations adjusted for all covariates was 1.00 (95%CI 0.97-1.04). The HR for severe exacerbations was marginally significant after adjustment for all covariates (HR 0.92, 95% CI 0.84 – 0.99).

Table 2. Hazard ratios (HR) for association of chronic obstructive pulmonary disease (COPD) exacerbation, urinary tract infection or death with statin use

Outcome	Covariates in model*	HR	(95% CI)	P
COPD exacerbation (N events=30,961)				
	Unadjusted	0.99	(0.94 - 1.04)	0.652
	Basic	0.99	(0.94 - 1.03)	0.583
	Basic+CVD	0.94	(0.90 - 0.99)	0.014
	Basic+COPD	1.03	(0.99 - 1.08)	0.168
	Basic+COPD+CVD	1.01	(0.96 - 1.06)	0.736
	Basic+COPD+CVD+comorbidities	1.01	(0.96 - 1.06)	0.657
COPD exacerbation, recurrent events (N events=106,961)				
	Unadjusted	1.02	(0.98 - 1.06)	0.359
	Basic	1.02	(0.98 - 1.06)	0.334
	Basic+CVD	0.94	(0.91 - 0.98)	0.002
	Basic+COPD	1.04	(1.01 - 1.08)	0.010
	Basic+COPD+CVD	1.00	(0.97 - 1.03)	0.999
	All covariates	1.00	(0.97 - 1.04)	0.885
Severe COPD exacerbation (N events=8,110)				
	Unadjusted	1.05	(0.97 - 1.14)	0.217
	Basic	1.00	(0.92 - 1.08)	0.977
	Basic+CVD	0.80	(0.74 - 0.87)	<0.001
	Basic+COPD	1.07	(0.99 - 1.16)	0.087
	Basic+COPD+CVD	0.90	(0.83 - 0.98)	0.016
	All covariates	0.92	(0.84 – 0.99)	0.034
Death from any cause (N events=5,355)				
	Unadjusted	0.87	(0.79 - 0.96)	0.005
	Basic	0.85	(0.77 - 0.93)	0.001
	Basic+CVD	0.58	(0.53 - 0.64)	<0.001
	Basic+COPD	0.88	(0.80 - 0.97)	0.009
	Basic+COPD+CVD	0.63	(0.57 - 0.69)	<0.001
	All covariates	0.63	(0.57 - 0.70)	<0.001
	Basic+COPD+Baseline CVD	0.83	(0.76 - 0.92)	<0.001
Urinary tract infection (N events=3,650)				
	Unadjusted	1.19	(1.07 - 1.33)	0.002
	Basic	1.28	(1.14 - 1.43)	<0.001

For peer review only - http://bmjopen.bmj.com/site/about/guidelines.xhtml

Basic+CVD	1.07	(0.95 - 1.20)	0.273
Basic+COPD	1.29	(1.16 - 1.44)	<0.001
Basic+COPD+CVD	1.09	(0.97 - 1.22)	0.159
All covariates	1.10	(0.98 - 1.23)	0.112

* Covariates included in the models. Basic adjustment, lifestyle and demographic variables including gender, age, IMD quintile (categorical), smoking status (never, ex, current); COPD, severity of chronic obstructive disease (COPD) at the index date including exacerbation frequency (categorised as 0,1,2,3,4,5+), severe exacerbation requiring hospitalisation, forced expiratory volume in one-second percent of predicted (categorised as 80, 50-<80, 30-<50, <30) , regular prescriptions for COPD-related medications, received oxygen; CVD, cardiovascular disease (CVD) related variables at index and as time-dependent variables including diabetes, hypertension, CVD diagnosis, body mass index (at index); Baseline CVD, CVD related variables at the index date (post hoc sensitivity analysis); All covariates, included Basic+COPD+CVD groups of covariates and also other comorbidities and health-related covariates recorded at index.

Table 3. Hazard ratios (HR) for association of chronic obstructive pulmonary disease (COPD) exacerbation, death or urinary tract infection with statin use. Follow-up was censored 90-days after the last statin prescription. HR are adjusted for all covariates.

Outcome	N events	HR	(95% CI)	P
COPD exacerbation	17,633	1.02	(0.94-1.09)	0.688
COPD exacerbation, recurrent events	102,342	1.00	(0.96-1.04)	0.995
Severe COPD exacerbation	4,365	0.89	(0.78-1.01)	0.080
Death	2,828	0.50	(0.42-0.60)	<0.001
Urinary tract infection	2,054	0.99	(0.82-1.20)	0.943

Secondary outcomes

Taking statins was associated with a lower risk of death from any cause. The HR after basic adjustment was 0.85 (95%CI 0.77 - 0.93) (Table 2). The association was considerably strengthened after adjusting for CVD risk factors. After adjustment for all covariates the HR was 0.63 (95% CI 0.57 - 0.70). After adjustment for all covariates but with CVD restricted to baseline values (post hoc sensitivity analysis) the HR was 0.83 (95% CI 0.76 - 0.92). There was an increased risk of UTI in people taking statins after basic adjustment (HR 1.28, 95%CI 1.14 - 1.43). This was reduced and no longer statistically significant after adjusting for all covariates (HR 1.10, 95%CI 0.98 - 1.23).

For peer review only - http://bmjopen.bmj.com/site/about/guidelines.xhtml

DISCUSSION

In this study of people with COPD, we did not find evidence to support a beneficial effect of statin use on incidence of COPD exacerbations. The hazard ratios were close to one whether we considered the first exacerbation or multiple exacerbations after study entry. Using statins was associated with a slightly lower (8%, 95% CI 1%-16%) risk of severe exacerbation requiring hospitalisation after adjusting for all covariates.

Our findings for the primary outcome of COPD exacerbation are consistent with those from randomised controlled trials[9 21 22], but contrast with those of previous observational studies which have found 30-40% lower risk of exacerbation with taking statins.[7 8] STATCOPE, the larger trial (885 participants) of simvastatin versus placebo for prevention of exacerbations among patients with moderate-severe COPD but no other indications for statin use was stopped early due to lack of benefit of statins on exacerbation rate.[22] The much smaller RODEO trial of rosuvastatin in patients with verified COPD but not CVD also failed to find any benefit of statin on exacerbation rate.[21] We did not restrict the population to a particular stage of COPD in our study, and patients with indications for statin treatment or previous CVD were not excluded. CVD is strongly linked to COPD,[23 24] and so some hospital admissions for severe exacerbation may have been attributable to CVD. This might explain the small beneficial effect that we observed of statin use on severe exacerbations.

We included UTI as a negative-control outcome. In other words, we would not expect an association with statin use if confounding was controlled for and if the study was free from other biases. We found that the rate of UTI was slightly increased in statin users, but this association became non-significant after adjustment for cardiovascular risk factors.

Taking statins was associated with a 15% (95% CI 7%-23%) lower risk of death after basic adjustment for demographic variables and a 37% lower risk of death (95% CI 30%-43%) after adjusting for all confounders. Our observed effect size is somewhat larger than has been estimated in trials for primary prevention in the general population, which is about 14%.[25 26] Healthy user biases, or the reverse in which COPD patients not on statins have poorly treated comorbid CVD which contributes to worse outcomes may have contributed to this observation.[12] Very ill patients with a higher risk of death or COPD exacerbation may not have been prescribed statins: patients in our study population who were prescribed statins had on average slightly milder COPD, and patients with end-stage COPD being managed palliatively are unlikely to be inititating a statin.

Those initiating statins had much higher prevalence of CVD-related variables at index date and incident over follow-up so we adjusted for time-dependent CVD. However, we may have

For peer review only - http://bmjopen.bmj.com/site/about/guidelines.xhtml

inadvertently biased the association between statins and death by including new CVD diagnoses after statin initiation, because CVD risk is also affected by statin exposure.[27] This raises the possibility that all HR that were adjusted for time-dependent CVD variables may have been subject to some degree of over-adjustment. The post hoc sensitivity analysis adjusting for baseline CVD resulted in an estimate of 17% (95% CI 8%-24%) lower risk of death, which is closer to expectations. However, this estimate is also likely to be biased because it does not allow for CVD developed after the index date.

Strengths and limitations of this study

Strengths of our study are the use of a large statin-naïve cohort with statin exposure included as a time-dependent exposure to minimise immortal time bias and prevalent user bias; adjustment for a wide range of potential confounding variables; and inclusion of control outcomes to investigate residual confounding.

The CPRD GOLD database is based on Primary Care data and therefore our study population is more generalizable to real-world clinical practice than selected populations recruited for clinical trials. CPRD GOLD has been used previously for a wide range of studies, and has been shown to be representative of the UK population and many variables extracted from the data have been validated externally including those related to COPD.[14 16 17]

Studies using databases of electronic health records also have their limitations. Our assessment of statin use was based on statin prescriptions and we cannot be certain that medications were dispensed or that patients were taking their medication. One study of individuals with COPD found that the proportion of prescriptions for COPD maintenance medications that were picked up was quite low as was adherence to these medications.[28] We learnt from discussions with PPI volunteers that some exacerbations might be treated in accident and emergency departments. However, even though these data are available it may be difficult to identify exacerbations in them, and no methodology has been validated yet, so by not including accident and emergency data we may have missed COPD exacerbations. Missing data is potentially an issue in electronic health record databases: in particular, we did not have an estimate of FEV_1 percent predicted for 28% of included patients.

Other limitations of this study relate to the use of an observational study design. People starting on statins are likely to be different from those not doing so. We included secondary outcomes to help to help to identify potential sources of bias. Nevertheless, we cannot be certain that there is not still residual confounding or other biases that we have not considered.

For peer review only - http://bmjopen.bmj.com/site/about/guidelines.xhtml

CONCLUSION

These data suggest that in people with COPD and no other indication, statins should not be prescribed in routine primary care to reduce COPD exacerbations. Our findings are consistent with the well-established benefit of statins for reducing death in patients with CVD risk factors, and confirm this in a COPD population.

FIGURE LEGEND

Figure 1 Flow chart describing the selection of the study population from the Clinical Practice Research Datalink (CPRD) GOLD database. FEV1/FVC is the ratio of forced expiratory volume in one second to forced vital capacity.

FUNDING

This study/project is funded by the National Institute for Health Research (NIHR) School for Primary Care Research (project reference 365). J Sheppard receives funding from the Wellcome Trust/Royal Society via a Sir Henry Dale Fellowship (ref: 211182/Z/18/Z). H Ashdown has been funded by an NIHR Doctoral Research Fellowship (DRF-2014-07-052).

COMPETING INTERESTS

C Butler reports grants from the NIHR and grants from the NIHR Health Protection Research Unit during the conduct of the study; personal fees from Pfizer outside of the submitted work.
J Sheppard reports grants from Wellcome Trust/Royal Society and from NIHR Oxford Biomedical Research Centre. H Ashdown has contributed to a COPD prescribing study using the CPRD and her academic department has received fees from Boehringer-Ingelheim for this work. C Bankhead reports grants from NIHR Oxford Biomedical Research Centre, grants from NIHR Applied Research Collaborative Oxford and Thames Valley during the conduct of the study.

AUTHOR CONTRIBUTIONS

M Smith planned and conducted the study, carried out statistical analyses and drafted the manuscript. H Ashdown provided clinical expertise on COPD. All authors had full access to all the data in the study, contributed to the study design, interpretation of results, revisions of the manuscript, and approved the final manuscript.

For peer review only - http://bmjopen.bmj.com/site/about/guidelines.xhtml

ACKNOWLEDGEMENTS

C Bankhead and M Smith are supported by the National Institute for Health Research (NIHR) Oxford Biomedical Research Centre (BRC). C Bankhead is supported by the NIHR Applied Research Collaborative Oxford and Thames Valley. J Sheppard is supported by an NIHR Oxford BRC Senior Fellowship The views expressed are those of the author(s) and not necessarily those of the NIHR or the Department of Health and Social Care. We thank the Patient and Public Involvement volunteers for their time and for sharing their knowledge.

ETHICS APPROVAL

The project protocol was approved by the CPRD Independent Scientific Advisory Committee (protocol number 18_059A).

DATA SHARING STATEMENT

This study is based on data from the CPRD obtained under licence from the UK Medicines and Healthcare products Regulatory Agency. Information on how to access the data used for these analyses is available at https://www.cprd.com/home/. The work uses data provided by patients and collected by the NHS as part of their care and support and would not have been possible without access to this data. The NIHR recognises and values the role of patient data, securely accessed and stored, both in underpinning and leading to improvements in research and care.

REFERENCES

1. Vogelmeier CF, Criner GJ, Martinez FJ, et al. Global Strategy for the Diagnosis, Management, and Prevention of Chronic Obstructive Lung Disease 2017 Report. GOLD Executive Summary. *American journal of respiratory and critical care medicine* 2017;195(5):557-82.

2. Divo M, Cote C, de Torres JP, et al. Comorbidities and risk of mortality in patients with chronic obstructive pulmonary disease. *American journal of respiratory and critical care medicine* 2012;186(2):155-61.

3. NICE. Cardiovascular disease: risk assessment and reduction, including lipid modification. *NICE guidelines [CG181]* Published date: July 2014 2014 Last updated: September 2016

4. Gan WQ, Man SFP, Senthilselvan A, et al. Association between chronic obstructive pulmonary disease and systemic inflammation: a systematic review and a meta-analysis. *Thorax* 2004;59(7):574-80.

For peer review only - http://bmjopen.bmj.com/site/about/guidelines.xhtml

5. Agusti A, Edwards LD, Rennard SI, et al. Persistent systemic inflammation is associated with poor clinical outcomes in COPD: a novel phenotype. *PloS one* 2012;7(5):e37483.
6. Quist-Paulsen P. Statins and inflammation: an update. *Current Opinion in Cardiology* 2010;25(4)
7. Cao C, Wu Y, Xu Z, et al. The effect of statins on chronic obstructive pulmonary disease exacerbation and mortality: a systematic review and meta-analysis of observational research. *Scientific reports* 2015;5:16461.
8. Ingebrigtsen TS, Marott JL, Nordestgaard BG, et al. Statin use and exacerbations in individuals with chronic obstructive pulmonary disease. *Thorax* 2015;70(1):33-40.
9. Walsh A, Perrem L, Khashan AS, et al. Statins versus placebo for people with chronic obstructive pulmonary disease. *The Cochrane database of systematic reviews* 2019;7(7):CD011959-CD59.
10. Suissa S. Immortal time bias in pharmaco-epidemiology. *American journal of epidemiology* 2008;167(4):492-9.
11. Danaei G, Tavakkoli M, Hernan MA. Bias in observational studies of prevalent users: lessons for comparative effectiveness research from a meta-analysis of statins. *American journal of epidemiology* 2012;175(4):250-62.
12. Young RP, Hopkins RJ, Agusti A. Statins as adjunct therapy in COPD: how do we cope after STATCOPE? *Thorax* 2014;69(10):891-4.
13. Herrett E, Gallagher AM, Bhaskaran K, et al. Data Resource Profile: Clinical Practice Research Datalink (CPRD). *Int J Epidemiol* 2015;44(3):827-36.
14. Quint JK, Müllerova H, DiSantostefano RL, et al. Validation of chronic obstructive pulmonary disease recording in the Clinical Practice Research Datalink (CPRD-GOLD). 2014;4(7):e005540.
15. Danese MD, Gleeson M, Kutikova L, et al. Management of lipid-lowering therapy in patients with cardiovascular events in the UK: a retrospective cohort study. *BMJ open* 2017;7(5):e013851-e51.
16. Rothnie KJ, Mullerova H, Hurst JR, et al. Validation of the Recording of Acute Exacerbations of COPD in UK Primary Care Electronic Healthcare Records. *PloS one* 2016;11(3):e0151357.
17. Rothnie KJ, Mullerova H, Thomas SL, et al. Recording of hospitalizations for acute exacerbations of COPD in UK electronic health care records. *Clin Epidemiol* 2016;8:771-82.
18. Mullerova H, Shukla A, Hawkins A, et al. Risk factors for acute exacerbations of COPD in a primary care population: a retrospective observational cohort study. *BMJ open* 2014;4(12):e006171.

For peer review only

For peer review only - http://bmjopen.bmj.com/site/about/guidelines.xhtml

19. Calverley PM, Tetzlaff K, Dusser D, et al. Determinants of exacerbation risk in patients with COPD in the TIOSPIR study. *International journal of chronic obstructive pulmonary disease* 2017;12:3391-405.

20. Amorim LD, Cai J. Modelling recurrent events: a tutorial for analysis in epidemiology. *Int J Epidemiol* 2014;44(1):324-33.

21. Neukamm A, Hoiseth AD, Einvik G, et al. Rosuvastatin treatment in stable chronic obstructive pulmonary disease (RODEO): a randomized controlled trial. *Journal of internal medicine* 2015;278(1):59-67.

22. Criner GJ, Connett JE, Aaron SD, et al. Simvastatin for the prevention of exacerbations in moderate-to-severe COPD. *The New England journal of medicine* 2014;370(23):2201-10.

23. Morgan AD, Zakeri R, Quint JK. Defining the relationship between COPD and CVD: what are the implications for clinical practice? *Therapeutic advances in respiratory disease* 2018;12:1753465817750524-24.

24. Rabe KF, Hurst JR, Suissa S. Cardiovascular disease and COPD: dangerous liaisons? *European respiratory review : an official journal of the European Respiratory Society* 2018;27(149)

25. Taylor F, Huffman MD, Macedo AF, et al. Statins for the primary prevention of cardiovascular disease. *Cochrane Database Syst Rev* 2013(1):CD004816.

26. Chou R, Dana T, Blazina I, et al. Statins for Prevention of Cardiovascular Disease in Adults: Evidence Report and Systematic Review for the US Preventive Services Task Force. *Jama* 2016;316(19):2008-24.

27. Mansournia MA, Etminan M, Danaei G, et al. Handling time varying confounding in observational research. *BMJ (Clinical research ed)* 2017;359:j4587.

28. Ingebrigtsen TS, Marott JL, Nordestgaard BG, et al. Low use and adherence to maintenance medication in chronic obstructive pulmonary disease in the general population. *J Gen Intern Med* 2015;30(1):51-59.

For peer review only

For peer review only - http://bmjopen.bmj.com/site/about/guidelines.xhtml

Figure 1. Selection of the study population

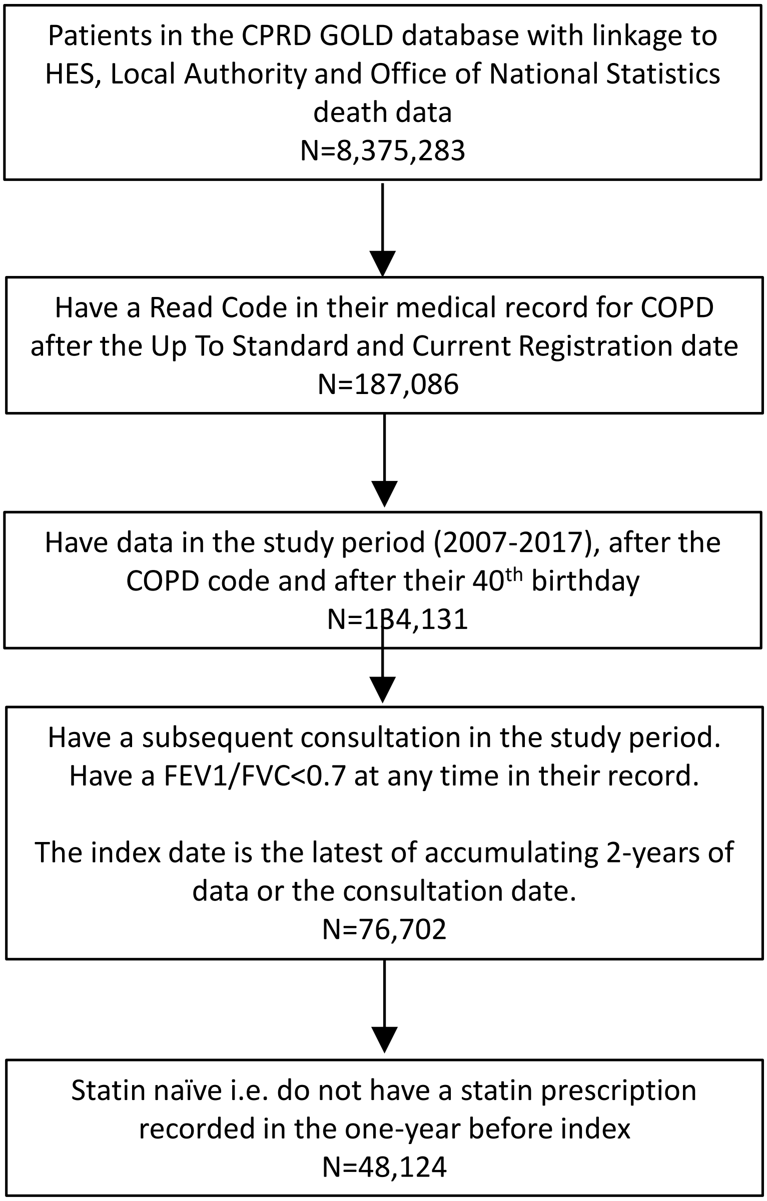

STROBE 2007 (v4) Statement—Checklist of items that should be included in reports of *cohort studies*

Section/Topic	Item #	Recommendation	Reported on page #
**Title and abstract**	1	(*a*) Indicate the study's design with a commonly used term in the title or the abstract	Title & Abstract
		(*b*) Provide in the abstract an informative and balanced summary of what was done and what was found	2-3
**Introduction**			
Background/rationale	2	Explain the scientific background and rationale for the investigation being reported	3-4
Objectives	3	State specific objectives, including any prespecified hypotheses	3
**Methods**			
Study design	4	Present key elements of study design early in the paper	4
Setting	5	Describe the setting, locations, and relevant dates, including periods of recruitment, exposure, follow-up, and data collection	4
Participants	6	(*a*) Give the eligibility criteria, and the sources and methods of selection of participants. Describe methods of follow-up	5
		(*b*) For matched studies, give matching criteria and number of exposed and unexposed	NA
Variables	7	Clearly define all outcomes, exposures, predictors, potential confounders, and effect modifiers. Give diagnostic criteria, if applicable	5
Data sources/ measurement	8*	For each variable of interest, give sources of data and details of methods of assessment (measurement). Describe comparability of assessment methods if there is more than one group	6
Bias	9	Describe any efforts to address potential sources of bias	4
Study size	10	Explain how the study size was arrived at	Fig 1
Quantitative variables	11	Explain how quantitative variables were handled in the analyses. If applicable, describe which groupings were chosen and why	7
Statistical methods	12	(*a*) Describe all statistical methods, including those used to control for confounding	6-7
		(*b*) Describe any methods used to examine subgroups and interactions	NA
		(*c*) Explain how missing data were addressed	7
		(*d*) If applicable, explain how loss to follow-up was addressed	5
		(*e*) Describe any sensitivity analyses	7
**Results**			

For peer review only

Participants	13*	(a) Report numbers of individuals at each stage of study—eg numbers potentially eligible, examined for eligibility, confirmed eligible, included in the study, completing follow-up, and analysed	Fig 1, p7
		(b) Give reasons for non-participation at each stage	Fig 1
		(c) Consider use of a flow diagram	Fig 1
Descriptive data	14*	(a) Give characteristics of study participants (eg demographic, clinical, social) and information on exposures and potential confounders	Table 1
		(b) Indicate number of participants with missing data for each variable of interest	Table 1
		(c) Summarise follow-up time (eg, average and total amount)	10
Outcome data	15*	Report numbers of outcome events or summary measures over time	10
Main results	16	(*a*) Give unadjusted estimates and, if applicable, confounder-adjusted estimates and their precision (eg, 95% confidence interval). Make clear which confounders were adjusted for and why they were included	Table 2
		(*b*) Report category boundaries when continuous variables were categorized	P7, Table 2
		(*c*) If relevant, consider translating estimates of relative risk into absolute risk for a meaningful time period	NA
Other analyses	17	Report other analyses done—eg analyses of subgroups and interactions, and sensitivity analyses	Table2, Table 3
**Discussion**			
Key results	18	Summarise key results with reference to study objectives	P12
**Limitations**			
Interpretation	20	Give a cautious overall interpretation of results considering objectives, limitations, multiplicity of analyses, results from similar studies, and other relevant evidence	P12-p13
Generalisability	21	Discuss the generalisability (external validity) of the study results	P12-P13
**Other information**			
Funding	22	Give the source of funding and the role of the funders for the present study and, if applicable, for the original study on which the present article is based	P14

*Give information separately for cases and controls in case-control studies and, if applicable, for exposed and unexposed groups in cohort and cross-sectional studies.

Note: An Explanation and Elaboration article discusses each checklist item and gives methodological background and published examples of transparent reporting. The STROBE checklist is best used in conjunction with this article (freely available on the Web sites of PLoS Medicine at http://www.plosmedicine.org/, Annals of Internal Medicine at http://www.annals.org/, and Epidemiology at http://www.epidem.com/). Information on the STROBE Initiative is available at www.strobe-statement.org.

For peer review only

BMJ Open

Statin prescription in patients with chronic obstructive pulmonary disease and risk of exacerbations: a retrospective cohort study in the Clinical Practice Research Datalink

Journal:	*BMJ Open*
Manuscript ID	bmjopen-2021-050757.R1
Article Type:	Original research
Date Submitted by the Author:	11-Aug-2021
Complete List of Authors:	Smith, Margaret; University of Oxford, Nuffield Department of Primary Care Health Sciences; Oxford University Hospitals NHS Foundation Trust, NIHR Oxford Biomedical Research Centre<br>Ashdown, Helen; University of Oxford, Nuffield Department of Primary Care Health Sciences<br>Sheppard, James; University of Oxford, Nuffield Department of Primary Care Health Sciences<br>Butler, Christopher C.; University of Oxford, Nuffield Department of Primary Health Care Sciences<br>Bankhead, Clare; University of Oxford, Nuffield Department of Primary Care Health Sciences; Oxford University Hospitals NHS Foundation Trust, NIHR Oxford Biomedical Research Centre
**Primary Subject Heading**:	Respiratory medicine
Secondary Subject Heading:	General practice / Family practice, Pharmacology and therapeutics
Keywords:	Chronic airways disease < THORACIC MEDICINE, Adult thoracic medicine < THORACIC MEDICINE, THERAPEUTICS

SCHOLARONE™
Manuscripts

I, the Submitting Author has the right to grant and does grant on behalf of all authors of the Work (as defined in the below author licence), an exclusive licence and/or a non-exclusive licence for contributions from authors who are: i) UK Crown employees; ii) where BMJ has agreed a CC-BY licence shall apply, and/or iii) in accordance with the terms applicable for US Federal Government officers or employees acting as part of their official duties; on a worldwide, perpetual, irrevocable, royalty-free basis to BMJ Publishing Group Ltd ("BMJ") its licensees and where the relevant Journal is co-owned by BMJ to the co-owners of the Journal, to publish the Work in this journal and any other BMJ products and to exploit all rights, as set out in our licence.

The Submitting Author accepts and understands that any supply made under these terms is made by BMJ to the Submitting Author unless you are acting as an employee on behalf of your employer or a postgraduate student of an affiliated institution which is paying any applicable article publishing charge ("APC") for Open Access articles. Where the Submitting Author wishes to make the Work available on an Open Access basis (and intends to pay the relevant APC), the terms of reuse of such Open Access shall be governed by a Creative Commons licence – details of these licences and which Creative Commons licence will apply to this Work are set out in our licence referred to above.

Other than as permitted in any relevant BMJ Author's Self Archiving Policies, I confirm this Work has not been accepted for publication elsewhere, is not being considered for publication elsewhere and does not duplicate material already published. I confirm all authors consent to publication of this Work and authorise the granting of this licence.

For peer review only

Statin prescription in patients with chronic obstructive pulmonary disease and risk of exacerbations: a retrospective cohort study in the Clinical Practice Research Datalink

Dr Margaret C Smith[1,2], Dr Helen Frances Ashdown[1], Dr James Peter Sheppard[1,2], Professor Christopher C Butler[1], Professor Clare Bankhead[1,2]

[1] Nuffield Department of Primary Care Health Sciences, University of Oxford, Oxford, UK

[2] NIHR Oxford Biomedical Research Centre, Oxford University Hospitals NHS Foundation Trust

Corresponding Author: Dr Margaret Smith,

Nuffield Department of Primary Care Health Sciences, University of Oxford, Radcliffe Primary Care Building, Radcliffe Observatory Quarter, Woodstock Road, Oxford, OX2 6GG.

Margaret.Smith@phc.ox.ac.uk

Word count of main text 3816:

Number of Tables: 3; Number of Figures: 1

Key words: chronic obstructive pulmonary disease, exacerbation, statin, death, urinary tract infection, cohort study

For peer review only - http://bmjopen.bmj.com/site/about/guidelines.xhtml

ABSTRACT

Objective

Observational studies have suggested a beneficial effect of taking statins on frequency of chronic obstructive pulmonary disease (COPD) exacerbations. However, clinical trials of statins in people with COPD did not confirm those results. This study aimed to investigate this association using a methodological approach, which reduces the biases associated with some previous observational study designs.

Design

Retrospective cohort study comparing new-users of statins with non-users.

Setting

General practices in England contributing to the Clinical Practice Research Datalink in 2007-2017, with linkage to data on Hospital Episode Statistics inpatient episodes.

Participants

48,124 people with COPD, aged over 40 years, who had not been prescribed statin in the previous year.

Exposure

Participants became new-users of statins at their first prescription for a statin during follow-up. They were then assumed to remain statin users. Statin users were compared with non-users.

Outcomes

Primary outcomes were COPD exacerbation, or severe exacerbation requiring hospitalisation. Secondary outcomes were death from any cause (for comparison with other studies) and urinary tract infection (negative-control). Maximum follow-up was 3 years. Adjusted hazard ratios (HR) were calculated using time-dependent Cox regression. The Andersen-Gill model was used for recurrent exacerbations. Covariates included demographic variables, variables related to COPD severity, cardiovascular comorbidities as time-dependent variables, and other comorbidities at baseline.

For peer review only

For peer review only - http://bmjopen.bmj.com/site/about/guidelines.xhtml

Results

7,266 participants became new-users of statins over an average 2.5 years of follow-up. In total 30,961 people developed an exacerbation, 8,110 severe exacerbation, 3,650 urinary tract infection and 5,355 died. Adjusted HR (95% CI) in statin users compared to non-users were first exacerbation 1.01 (0.97-1.06), severe exacerbation 0.92 (0.85-1.00), number of exacerbations 1.00 (0.97-1.04), urinary tract infection 1.10 (0.98-1.24) and death 0.63 (0.57-0.70).

Conclusions

In this study of health records from a Primary Care database statin use in people with COPD was not associated with a lower risk of COPD exacerbation.

(300 words)

For peer review only

STRENGTHS AND LIMITATIONS OF THIS STUDY

- We used a large real-world cohort of over 48,000 people with COPD who were statin naïve at study entry.
- The exposure was defined as new statin prescription in follow-up and analysed as a time-dependent variable. New user designs are less susceptible to prevalent-user bias.
- We included secondary outcomes and various levels of confounding adjustment to investigate some potential sources of bias.
- This study identified statin users from their first prescripton of statins. We cannot be certain that the medication was taken and adherence may have declined over follow-up.
- As this was an observational study, we cannot be certain that residual confounding or other biases did not affect our results

INTRODUCTION

Chronic obstructive pulmonary disease (COPD) is characterised by progressive and irreversible airflow limitation, together with sudden flare-ups or exacerbations. Frequent exacerbations accelerate loss of lung function and are associated with poorer quality of life and poor survival.[1] COPD is often associated with the presence of other co-morbidities, of which cardiovascular disease (CVD) is one of the most significant.[2] Therefore many people with COPD are likely to be

For peer review only - http://bmjopen.bmj.com/site/about/guidelines.xhtml

recommended medication for primary or secondary prevention of CVD, including with statins.[3] Increased levels of biomarkers reflecting systemic inflammation are also part of the pathology of COPD. Levels of inflammatory biomarkers increase during the acute phase of an exacerbation, and in individuals with stable COPD elevated levels of these biomarkers are associated with higher frequency of exacerbations.[4 5] Statins have been found to have anti-inflammatory effects, as well as reducing cholesterol.[6] Therefore there is a theoretical possibility that statins may have a beneficial role in COPD management over and above reducing cardiovascular risk.

Several observational studies have found an association between statin use and fewer exacerbations in COPD patients.[7 8] However randomised controlled trials have failed to confirm any such associations.[9] Several explanations for the conflict of trial results with those from observational studies have been proposed. Firstly, immortal time bias arises from misspecification of exposures so that there is a span of cohort follow-up during which, because of exposure definition, the outcome under study cannot occur.[10] Secondly, prevalent user bias occurs when risk of an outcome changes over time in a population exposed to a drug treatment.[11] Lastly, the population and treatment in the clinical trials were very precisely defined and unlikely to be typical of the general population.[12]

A study design in which all patients are statin naïve at study entry, and in which initiation of statins is included as a time dependent variable can reduce the likelihood of some of these biases. This study aimed to use such a study design to examine the association between statin prescription and incidence of COPD exacerbations in a general population of patients attending routine primary care.

METHODS

Study design

The study is a retrospective cohort study comparing new-users of statins with non-users. It was conducted within the Clinical Practice Research Datalink (CPRD) GOLD database, which is a collection of anonymised primary care records from ~700 UK practices. These data have been shown to be representative of the general UK population in terms of age, sex and ethnicity.[13] We used data from the three-quarters of CPRD English practices with linkage to the English Hospital Episode Statistics (HES), and mortality data from the UK Office for National Statistics (ONS), and patient-level social deprivation indices.

For peer review only - http://bmjopen.bmj.com/site/about/guidelines.xhtml

For peer review only

Patient and Public Involvement

As this is a retrospective database study there are limited opportunities for Patient and Public Involvement in study design or conduct. During the development of the protocol, we discussed experience of exacerbations with three people who had COPD themselves or had experience of caring for people with COPD.

Population and exposure

We included patients aged at least 40 years, who consulted between Jan 2007 and July 2017, and had a preceding Read code for COPD in their medical records within research-quality data. Research quality data fulfils basic CPRD-defined quality criteria at the practice (recorded death rates are within a pre-defined range based on the UK average and that there is continuity in recording of deaths and other events) and patient level (permanently registered at a practice with a valid birth year and registration date) level.[14] To confirm the presence of COPD, a forced expiratory volume in one second/forced vital capacity (FEV1/FVC) of <0.7 had to also be recorded.[15] To ensure that patients were actively consulting and that there were enough data to identify baseline values of covariates, the index date was defined as the latest of the consultation date and the date of accumulating two years of research quality data in the current practice. Patients in the selected cohort were also all statin naïve as they were only included in the cohort if there had been no statin prescription in the 12 months before the index date. They were defined as newly prescribed users of statins once a prescription was issued on the index date or during follow-up.

Outcomes

Patients were followed-up until they were censored after 3-years, or until the end of the study period or they died or left their practice or their practice stopped contributing data to CPRD GOLD, if any of these occurred within 3-years. Follow-up was limited to 3 years because we expected adherence to statins to drop-off over time.[16]

Our primary outcomes were any COPD exacerbation and severe exacerbation requiring hospitalisation. An acute exacerbation of COPD in the Primary Care record was defined as a code for acute exacerbation, code for lower respiratory tract infection, prescriptions for 5-14 days of exacerbation-specific antibiotics and oral steroid on the same date, or a symptom of exacerbation together with a prescription for exacerbation-specific antibiotics or oral steroids.[17] Exacerbations were also identified from the HES inpatient record, from a COPD or acute respiratory codes as the primary diagnosis of hospitalisation (ICD-10 J00, J06, J09-18, J20-22, J40-44, J96), or from a code for acute exacerbation of COPD anywhere in the hospitalisation record (J44.0, J44.1).[18] We identified all

For peer review only - http://bmjopen.bmj.com/site/about/guidelines.xhtml

exacerbations in follow-up. To prevent double counting, events indicating exacerbations within 14 days of each other were assumed to describe the same exacerbation, with the exacerbation date taken from the first code. A severe exacerbation was any exacerbation with a relevant code, as described above, in the HES inpatient data at some point in its duration.

Secondary outcomes were death from any cause, and urinary tract infection (UTI). Death was chosen so that we could compare the observed association with results from randomised controlled trials in the general population. UTI recorded in Primary Care data was included as a negative-control outcome because we did not expect any association with statin use. A strong association between statin use and UTI might indicate some bias in the study design.

Covariates

We assessed potential confounders at the index date and time dependently. For the purpose of adjustment in regression analyses covariates were combined into groups: lifestyle and demographic factors, markers of COPD severity, CVD-related variables, and other co-morbidities or health-related variables.

- Lifestyle and demographic variables (basic adjustment) included gender, age at index, deprivation (as quintile of Index of Multiple Deprivation), and smoking status before index.
- Covariates related to COPD severity were FEV_1 percent of predicted, number of COPD exacerbations, severe exacerbation requiring hospitalisation, regular prescription of COPD-related medication (short-acting bronchodilator, long-acting bronchodilator, inhaled corticosteroid, nebuliser), and receiving oxygen therapy.[19 20] The most recent FEV_1 percent of predicted in the two years prior to index was extracted. All other variables were assessed in the one-year before index. COPD exacerbations were defined as for outcomes. Regular prescription was defined as at least three prescriptions in the year.
- CVD-related variables were previous CVD, diabetes and hypertension. These variables were assessed as if they had occurred ever before index and time-dependently during follow-up. We included this group of variables as time-dependent covariates in main analyses because they are very likely to be associated with statin exposure. CVD was defined as a diagnosis code for one of coronary heart disease, stroke, heart failure or peripheral artery disease in the Primary Care data or in the linked HES inpatient data. Hypertension was defined as a diagnosis code or a prescription for an antihypertensive medication. We also included body mass index (BMI) (the most recent measurement in two years before index) in this group of covariates.

For peer review only - http://bmjopen.bmj.com/site/about/guidelines.xhtml

- Comorbidities and other health-related variables including asthma, other respiratory disease (bronchiectasis, cystic fibrosis, alpha-1 anti-trypsin-deficiency, and interstitial lung disease), lung cancer, rheumatoid arthritis, gastro-oesophageal reflux disease and osteoporosis were assessed from codes in Primary Care and HES inpatient data recorded at any time before the index date. Depression, influenza vaccination, and number of doctor or nurse consultations were assessed in Primary Care data in the year before index.

Statistical Analysis

We used Cox regression to investigate the association of statin use with time to first event for exacerbation, severe exacerbation, death or UTI. The proportional hazards assumption was assessed for the time-independent covariates using graphical methods (-ln –ln of survival curves). For analyses of recurrent exacerbation we used the Andersen and Gill model.[21] Statin use was defined as a binary time-dependent variable. All patients were non-users of statin immediately before the index date. They became new-users of statins at their first prescription of statin on the index date or during follow-up. In main analyses, we took an intention to treat approach in which people were assumed to remain statin users from the initial prescription until they were censored after a maximum of 3-years of follow-up. We censored after 3-years because we expected adherence to statins to fall over time, which could dilute the observed effect size. The analyses were repeated with combinations of the different sets of covariates to try to understand the role of confounding. All analyses were conducted in Stata 16 (StataCorp, Texas, US).

Age at index and BMI were included in analyses as continuous covariates. FEV_1 percent of predicted was included as a categorical variable (≥80%, 50-<80%, 30-<50%, <30%). Quintile of IMD, number of exacerbations (0, 1, 2, 3, 4, ≥5) and smoking status (never, ex, current) were also included as categorical variables. Other covariates were included as binary variables. Time dependent variables were incorporated by splitting patient records at the date of the first prescription for statin or when CVD, diabetes or hypertension were first recorded.

Missing values of BMI or FEV_1 percent of predicted were imputed with multiple imputation by chained equations, using a linear regression model for imputing FEV_1 percent of predicted and predictive mean matching for BMI, and including all variables to be included in planned analyses (including outcomes). Thirty imputed data sets were calculated and estimates from regressions were pooled using Rubin's rules. People with missing smoking data were assumed non-smokers. Missing values of other variables were interpreted as a null value e.g. no prescription or no comorbidity. We

For peer review only - http://bmjopen.bmj.com/site/about/guidelines.xhtml

also repeated analyses restricted to people with complete data. We did not find any substantive difference in results (data not shown).

In secondary analyses, we censored 90-days after the last recorded statin prescription to investigate the effect of non-adherence on associations. In main analyses that were adjusted for CVD-related variables we adjusted for these variables at index and time-dependently. A *post hoc* sensitivity analysis was also conducted for the death outcome in which we adjusted for CVD variables assessed at baseline rather than the time-dependent CVD variables.

RESULTS

Baseline characteristics

A total of 8,375,283 people were available with complete linkage within the CPRD GOLD database (Figure 1). Within these, we identified 76,702 people aged 40+ years who fulfilled the definition of a COPD diagnosis, had a recent record of consulting their GP and had data in the study period. Of these, 48,124 were statin naïve and were included in the cohort. Of these, 7,266 had at least one statin prescription on the index date or subsequently during a maximum of 3-years of follow-up. For 40,858 there was no record of a statin prescription in follow-up.

People who subsequently started on statins i.e. became new-users of statins, had different baseline characteristics compared to those who did not (Table 1). They were less likely to be female (42% compared to 50%). They were also more likely to have social deprivation above the median (57% vs. 54%) but slightly less likely to be current smokers (8% vs. 10%). Variables related to COPD severity indicated that COPD was on average slightly less severe at baseline in the new statin users. Those who became statin users had a slightly higher body mass index at baseline (27 vs 26 kgm^{-2}) and considerably higher prevalence of hypertension (50% vs 39%), CVD (14% vs 8%) and diabetes (6% vs 2%) at baseline. More had hypertension, CVD or diabetes newly recorded in follow-up. New statin-users were also more like to have an influenza vaccination in the year before baseline (72% vs 66%), and more likely to make 5+ visits to a GP (57% vs 55%) or nurse (24% vs 22%).

For peer review only - http://bmjopen.bmj.com/site/about/guidelines.xhtml

Table 1. Descriptive statistics for the study population according to whether they received a statin prescription in follow-up i.e. became new-users of statins. Baseline characteristics are given for all variables. New cases over follow-up are also given for cardiovascular disease and related conditions. Statistics are n (%) unless otherwise stated.

	Prescription for statin in follow-up	
**Variable**	**No (N=40858)**	**Yes (N=7266)**
Index date (median)	March 2008	July 2007
**Lifestyle and demographic**		
Female	20375 (50%)	3045 (42%)
Mean(SD) age (years)	67(12)	68(10)
Deprivation above median (IMD 2007)	22109 (54%)	4174 (57%)
Smoking status		
Current	4153 (10%)	604 (8%)
Ex	19978 (49%)	3661 (50%)
Never	16524 (40%)	2973 (41%)
Missing	203 (0%)	28 (0%)
**Related to COPD severity**		
Mean(SD) FEV_1 (percent of predicted)	63(21)	65(21)
Missing	11278 (28%)	2062 (28%)
FEV_1 percent of predicted		
≥ 80	6073 (15%)	1123 (15%)
≥ 50 - < 80	15152 (37%)	2788 (38%)
≥ 30 - < 50	6966 (17%)	1100 (15%)
< 30	1389 (3%)	193 (3%)
Missing	11278 (28%)	2062 (28%)
Number of COPD exacerbations in 1-year before index		
0	20336 (50%)	3785 (52%)
1	11154 (27%)	1914 (26%)
2	5073 (12%)	884 (12%)
3	2237 (5%)	346 (5%)
4	1024 (3%)	175 (2%)
≥5	1034 (3%)	162 (2%)
At least one severe exacerbation in 1-year before index	2981 (7%)	499 (7%)
Regular prescription (3+) of COPD medication in 1-year before index		
Short acting bronchodilator	20936 (51%)	3661 (50%)
Long acting bronchodilator	18068 (44%)	3075 (42%)

For peer review only

Inhaled corticosteroid	19136 (47%)	3323 (46%)
Nebuliser	1654 (4%)	230 (3%)
On oxygen therapy	423 (1%)	49 (1%)
**Related to cardiovascular disease**		
Diabetes		
Recorded before index date	1007 (2%)	462 (6%)
New over follow-up	482 (1%)	693 (10%)
Hypertension		
Recorded before index date	16093 (39%)	3661 (50%)
New over follow-up	4632 (11%)	2066 (28%)
Cardiovascular disease		
Recorded before index date	3241 (8%)	991 (14%)
New over follow-up	1812 (4%)	1984 (27%)
Body mass index (kg m^{-2}) Mean(SD)	26(6)	27(6)
Missing	10838 (27%)	1679 (23%)
**Comorbidities and other health-related recorded before index date**		
Respiratory comorbidities		
Asthma	10818 (26%)	1890 (26%)
Other respiratory disease	1289 (3%)	195 (3%)
Other comorbidities		
Lung cancer	331 (1%)	46 (1%)
Depression in 1-year before index	2808 (7%)	474 (7%)
Rheumatoid arthritis	700 (2%)	123 (2%)
Gastro-oesophageal reflux disease	4966 (12%)	995 (14%)
Osteoporosis	2113 (5%)	300 (4%)
Influenza vaccination in 1-year before index	27143 (66%)	5211 (72%)
Regular (5+) contacts with practice staff in 1-year before index		
GP	22525 (55%)	4153 (57%)
Nurse	8979 (22%)	1772 (24%)

SD, standard deviation; IMD, index of multiple deprivation, COPD chronic obstructive pulmonary disease; FEV_1, forced expiratory volume in one second percent of predicted; GP, general practitioner

Associations of statin use with outcomes

The mean follow-up was 2.46 years. 17,350 (36%) people were censored before the end of the maximum 3 years of follow-up, due to death (5,355 (11%)), end of the study period (1,973 (4%)), or

For peer review only - http://bmjopen.bmj.com/site/about/guidelines.xhtml

leaving their current GP practice or the end of downloaded data in CPRD from their practice (10,022 (21%)). Over follow-up, there were 106,961 exacerbations recorded. 30,961 (64%) people had at least one exacerbation, 8,110 (17%) were hospitalised for a severe exacerbation and 3,650 (8%) had at least one UTI diagnosed. The mean follow-up in nonusers of statins (to censoring, death or starting on statins) was 2.23 years (total 107039 years). The mean follow-up after starting to use statins was 1.56 years (total 11307 person years.

The first statin prescription was simvastatin in 6,089 (84%) of the new users. It was atorvastatin for 989 (14%), pravastatin for 133 (2%), rosuvastatin for 53 (1%) and fluvastatin for two people. The number of statin prescriptions per person was 9 (4-18) (median and interquartile range). Amongst those whose first prescription was for simvastatin, 397(7%) had a prescription for a daily dose of 10mg, 1822 (30%) for 20mg, 3864 (64%) for 40mg and 6 (<1%) for 80mg.

COPD exacerbation

There was no association between statins and COPD exacerbation after adjustment for basic demographic variables (hazard ratio (HR) 0.99, 95%CI 0.94-1.03) (Table 2). Additionally adjusting for cardiovascular risk factors resulted in a small statistically significant beneficial effect of statins (HR 0.94, 95%CI 0.90-0.99) (Table 2). The association was constant after further adjustment for variables related to severity of COPD (HR 1.01, 95%CI 0.96-1.06). This remained the case after adjustment for all covariates (HR 1.01, 95%CI 0.96-1.06) and in analyses censoring 90-days after the last statin prescription (Table 3). A similar pattern of HR with different adjustments was seen for the recurrent COPD exacerbation outcome and for severe COPD exacerbations. The HR for recurrent COPD exacerbations adjusted for all covariates was 1.00 (95%CI 0.97-1.04). The HR for severe exacerbations was marginally significant after adjustment for all covariates (HR 0.92, 95% CI 0.84 – 0.99).

Table 2. Hazard ratios (HR) for association of chronic obstructive pulmonary disease (COPD) exacerbation, urinary tract infection or death with statin use

Outcome	Covariates in model*	HR	(95% CI)	P
Primary outcomes				
COPD exacerbation (N events in statin users, non-users= 1893, 29068)				
	Unadjusted	0.99	(0.94 - 1.04)	0.652
	Basic	0.99	(0.94 - 1.03)	0.583
	Basic+CVD	0.94	(0.90 - 0.99)	0.014
	Basic+COPD	1.03	(0.99 - 1.08)	0.168
	Basic+COPD+CVD	1.01	(0.96 - 1.06)	0.736

For peer review only - http://bmjopen.bmj.com/site/about/guidelines.xhtml

All covariates	1.01	(0.96 - 1.06)	0.657
COPD exacerbation, recurrent events (N events in statin users, non-users== 10352, 96609)			
Unadjusted	1.02	(0.98 - 1.06)	0.359
Basic	1.02	(0.98 - 1.06)	0.334
Basic+CVD	0.94	(0.91 - 0.98)	0.002
Basic+COPD	1.04	(1.01 - 1.08)	0.010
Basic+COPD+CVD	1.00	(0.97 - 1.03)	0.999
All covariates	1.00	(0.97 - 1.04)	0.885
Severe COPD exacerbation (N events in statin users, non-users= = 722, 7388)			
Unadjusted	1.05	(0.97 - 1.14)	0.217
Basic	1.00	(0.92 - 1.08)	0.977
Basic+CVD	0.80	(0.74 - 0.87)	<0.001
Basic+COPD	1.07	(0.99 - 1.16)	0.087
Basic+COPD+CVD	0.90	(0.83 - 0.98)	0.016
All covariates	0.92	(0.84 – 0.99)	0.034
Secondary outcomes			
Death from any cause (N events in statin users, non-users= = 466, 4889)			
Unadjusted	0.87	(0.79 - 0.96)	0.005
Basic	0.85	(0.77 - 0.93)	0.001
Basic+CVD	0.58	(0.53 - 0.64)	<0.001
Basic+COPD	0.88	(0.80 - 0.97)	0.009
Basic+COPD+CVD	0.63	(0.57 - 0.69)	<0.001
All covariates	0.63	(0.57 - 0.70)	<0.001
Basic+COPD+Baseline CVD	0.83	(0.76 - 0.92)	<0.001
Urinary tract infection (N events in statin users, non-users= 360, 3290)			
Unadjusted	1.19	(1.07 - 1.33)	0.002
Basic	1.28	(1.14 - 1.43)	<0.001
Basic+CVD	1.07	(0.95 - 1.20)	0.273
Basic+COPD	1.29	(1.16 - 1.44)	<0.001
Basic+COPD+CVD	1.09	(0.97 - 1.22)	0.159
All covariates	1.10	(0.98 - 1.23)	0.112

* Covariates included in the models. Basic adjustment, lifestyle and demographic variables including gender, age, IMD quintile (categorical), smoking status (never, ex, current); COPD, severity of chronic obstructive disease (COPD) at the index date including exacerbation frequency (categorised as 0,1,2,3,4,5+), severe exacerbation requiring hospitalisation, forced expiratory volume in one-second percent of predicted (categorised as 80, 50-<80, 30-<50, <30) , regular prescriptions for COPD-related medications, received oxygen; CVD, cardiovascular disease (CVD) related variables at index and as time-dependent variables including diabetes, hypertension, CVD diagnosis, body mass index (at index); All covariates, included Basic+COPD+CVD groups of covariates and also other comorbidities and health-related covariates recorded at index (see list in Methods). Baseline CVD,

For peer review only - http://bmjopen.bmj.com/site/about/guidelines.xhtml

For peer review only

CVD related variables at the index date (post hoc sensitivity analysis conducted for the death outcome only).

Table 3. Hazard ratios (HR) for association of chronic obstructive pulmonary disease (COPD) exacerbation, death or urinary tract infection with statin use. Follow-up was censored 90-days after the last statin prescription. HR are adjusted for all covariates*.

Outcome	N events	HR	(95% CI)	P
Primary outcomes				
COPD exacerbation	17,633	1.02	(0.94-1.09)	0.688
COPD exacerbation, recurrent events	102,342	1.00	(0.96-1.04)	0.995
Secondary outcomes				
Severe COPD exacerbation	4,365	0.89	(0.78-1.01)	0.080
Death	2,828	0.50	(0.42-0.60)	<0.001
Urinary tract infection	2,054	0.99	(0.82-1.20)	0.943

* Covariates included in the models. Variables included in basic adjustment; variables related to severity of chronic obstructive disease (COPD) at the index date; cardiovascular disease related variables at index and as time-dependent variables; other comorbidities and health-related covariates recorded at index. See Methods and legend to Table 2 for details.

Secondary outcomes

Taking statins was associated with a lower risk of death from any cause. The HR after basic adjustment was 0.85 (95%CI 0.77 - 0.93) (Table 2). The association was considerably strengthened after adjusting for baseline and time-dependent CVD risk factors. After adjustment for all covariates the HR was 0.63 (95% CI 0.57 - 0.70). In the post hoc sensitivity analysis in which adjustment was for all covariates but with CVD restricted to baseline values the HR was 0.83 (95% CI 0.76 - 0.92). There was an increased risk of UTI in people taking statins after basic adjustment (HR 1.28, 95%CI 1.14 - 1.43). This was reduced and no longer statistically significant after adjusting for all covariates (HR 1.10, 95%CI 0.98 - 1.23).

DISCUSSION

In this study of people with COPD who were followed up for a maximum of 3-years, we did not find evidence to support a beneficial effect of statin use on incidence of COPD exacerbations. The hazard ratios were close to one whether we considered the first exacerbation or multiple exacerbations

For peer review only - http://bmjopen.bmj.com/site/about/guidelines.xhtml

after study entry. Using statins was associated with a slightly lower (8%, 95% CI 1%-16%) risk of severe exacerbation requiring hospitalisation after adjusting for all covariates.

Our findings for the primary outcome of COPD exacerbation are consistent with those from most randomised controlled trials[9 22 23], but contrast with those of previous observational studies which have found 30-40% lower risk of exacerbation with taking statins.[7 8] STATCOPE, the larger trial (885 participants) of simvastatin versus placebo for prevention of exacerbations among patients with moderate-severe COPD but no other indications for statin use was stopped early due to lack of benefit of statins on exacerbation rate.[23] The much smaller RODEO trial of rosuvastatin in patients with verified COPD but not CVD also failed to find any benefit of statin on exacerbation rate.[22] Recently published results from another small trial do however indicate some benefit of statin treatment on COPD exacerbation and mortality.[24] An explanation put forward by the authors for these results is that the study population was nearer "real world" than in previous trials as it included patients with subclinical cardiovascular disease. CVD is strongly linked to COPD, [25 26] and so some hospital admissions for severe exacerbation may have been attributable to CVD. The beneficial effect of statins may have arisen as a knock-on effect on severity of COPD exacerbations via reduced incidence of CVD. Observational studies and randomised controlled trials have found possible beneficial effects of statin use on development of many other diseases but as with statins and COPD exacerbations, most of these findings need further rigorous investigation.[27 28]

We included UTI as a negative-control outcome. In other words, we would not expect an association with statin use if confounding was controlled for and if the study was free from other biases. We found that the rate of UTI was slightly increased in statin users, but this association became non-significant after adjustment for cardiovascular risk factors.

Taking statins was associated with a 15% (95% CI 7%-23%) lower risk of death after basic adjustment for demographic variables and a 37% lower risk of death (95% CI 30%-43%) after adjusting for all confounders. Our observed effect size is somewhat larger than has been estimated in trials conducted in the general population, which is about 10%.[27 29] Healthy user biases, or the reverse in which COPD patients not on statins have poorly treated comorbid CVD which contributes to worse outcomes may have contributed to our observation.[12] Very ill patients with a higher risk of death or COPD exacerbation may not have been prescribed statins: patients in our study population who were prescribed statins had on average slightly milder COPD, and patients with end-stage COPD being managed palliatively are unlikely to be initiating a statin.

Those initiating statins had much higher prevalence of CVD-related variables at index date and incident over follow-up so we adjusted for time-dependent CVD. However, we may have

For peer review only - http://bmjopen.bmj.com/site/about/guidelines.xhtml

inadvertently biased some associations by including new CVD diagnoses after statin initiation, because CVD risk is also affected by statin exposure.[30] Therefore time-dependent CVD might also be a mediator on the path between statin use and outcomes. This raises the possibility that all HR that were adjusted for time-dependent CVD variables may have been subject to some degree of over-adjustment. The post hoc sensitivity analysis of the death outcome adjusting for baseline CVD but not time-dependent CVD resulted in an estimate of 17% (95% CI 8%-24%) lower risk of death, which is closer to expectations. However, this estimate is also likely to be biased because it does not allow for confounding by CVD developed after the index date.

Strengths and limitations of this study

Strengths of our study are the use of a large statin-naïve cohort with statin exposure included as a time-dependent exposure to minimise immortal time bias and prevalent user bias; adjustment for a wide range of potential confounding variables; and inclusion of control outcomes to investigate residual confounding.

The CPRD GOLD database is based on Primary Care data and therefore our study population is more generalizable to real-world clinical practice than selected populations recruited for clinical trials. CPRD GOLD has been used previously for a wide range of studies, and has been shown to be representative of the UK population and many variables extracted from the data have been validated externally including those related to COPD.[15 17 18]

Studies using databases of electronic health records also have their limitations. Non-adherence to prescribed statin medications or use of over-the-counter statins could potentially have reduced the size of any observed effect of statins on outcomes. Our assessment of statin use was based on statin prescriptions made in Primary Care. We cannot be certain that medications were dispensed or that patients were taking their medication. One study of individuals with COPD found that the proportion of prescriptions for COPD maintenance medications that were picked up was quite low as was adherence to these medications.[31] Furthermore low-dose statins have been available over-the-counter in the United Kingdom since these 2004, and these would not be recorded in Primary Care records. Over-the-counter statin medication is limited to 10mg simvastatin but most Primary Care prescriptions for statins in our study were for 40mg simvastatin. In addition, the support for over-the-counter statins by pharmacists and clinicians has been limited, and the take-up has been very low.[32 33]

We learnt from discussions with PPI volunteers that some exacerbations might be treated in accident and emergency departments. However, even though these data are available it may be

For peer review only - http://bmjopen.bmj.com/site/about/guidelines.xhtml

difficult to identify exacerbations in them, and no methodology has been validated yet, so by not including accident and emergency data we may have missed COPD exacerbations, although documentation should be sent to the patient's GP. Missing data is potentially an issue in electronic health record databases: in particular, we did not have an estimate of FEV_1 percent predicted for 28% of included patients.

Other limitations of this study relate to the use of an observational study design. People starting on statins are likely to be different from those not doing so. We included a wide range of confounding variables in our models, nevertheless, we cannot be certain that there is not still some residual confounding. As discussed above, we included variables related to CVD as time-dependent covariates, which may have led to some degree of over-adjustment. We included secondary outcomes to help to identify potential sources of bias, but we cannot be certain that our results are not affected by other sources of bias that we have not considered.

CONCLUSION

We did not find evidence to support an association between statin prescription and reduced risk of exacerbation in people with COPD. These data suggest that in people with COPD and no other indication, statins should not be prescribed in routine primary care to reduce COPD exacerbations. Our findings are consistent with the well-established benefit of statins for reducing death in patients with CVD risk factors, and confirm this in a COPD population.

FIGURE LEGEND

Figure 1 Flow chart describing the selection of the study population from the Clinical Practice Research Datalink (CPRD) GOLD database. FEV1/FVC is the ratio of forced expiratory volume in one second to forced vital capacity.

FUNDING

This study/project is funded by the National Institute for Health Research (NIHR) School for Primary Care Research (project reference 365). J Sheppard receives funding from the Wellcome Trust/Royal Society via a Sir Henry Dale Fellowship (ref: 211182/Z/18/Z). H Ashdown has been funded by an NIHR Doctoral Research Fellowship (DRF-2014-07-052).

For peer review only - http://bmjopen.bmj.com/site/about/guidelines.xhtml

COMPETING INTERESTS

C Butler reports grants from the NIHR and grants from the NIHR Health Protection Research Unit during the conduct of the study; personal fees from Pfizer outside of the submitted work. J Sheppard reports grants from Wellcome Trust/Royal Society and from NIHR Oxford Biomedical Research Centre. H Ashdown has contributed to a COPD prescribing study using the CPRD and her academic department has received fees from Boehringer-Ingelheim for this work. C Bankhead reports grants from NIHR Oxford Biomedical Research Centre, grants from NIHR Applied Research Collaborative Oxford and Thames Valley during the conduct of the study.

AUTHOR CONTRIBUTIONS

M Smith planned and conducted the study, carried out statistical analyses and drafted the manuscript. H Ashdown provided clinical expertise on COPD. M Smith, H Ashdown, J Sheppard, C Butler, C Bankhead had full access to all the data in the study, contributed to the study design, interpretation of results, revisions of the manuscript, and approved the final manuscript.

ACKNOWLEDGEMENTS

C Bankhead and M Smith are supported by the National Institute for Health Research (NIHR) Oxford Biomedical Research Centre (BRC) and by the NIHR Applied Research Collaborative Oxford and Thames Valley. J Sheppard is supported by an NIHR Oxford BRC Senior Fellowship. The views expressed are those of the author(s) and not necessarily those of the NIHR or the Department of Health and Social Care. We thank the Patient and Public Involvement volunteers for their time and for sharing their knowledge.

ETHICS APPROVAL

CPRD has ethics approval from the Health Research Authority to support research using anonymised patient data. GP practices choose to share patient data (primary care data) with CPRD for public health research purposes. However, individual patients can opt out of sharing their data with CPRD. The project protocol was approved by the CPRD Independent Scientific Advisory Committee (protocol number 18_059A).

DATA SHARING STATEMENT

This study is based on data from the CPRD obtained under licence from the UK Medicines and Healthcare products Regulatory Agency. Information on how to access the data used for these analyses is available at https://www.cprd.com/home/. The work uses data provided by patients and collected by the NHS as part of their care and support and would not have been possible without

For peer review only

access to this data. The NIHR recognises and values the role of patient data, securely accessed and stored, in both underpinning and leading to improvements in research and care.

REFERENCES

1. Vogelmeier CF, Criner GJ, Martinez FJ, et al. Global Strategy for the Diagnosis, Management, and Prevention of Chronic Obstructive Lung Disease 2017 Report. GOLD Executive Summary. *American journal of respiratory and critical care medicine* 2017;195(5):557-82.
2. Divo M, Cote C, de Torres JP, et al. Comorbidities and risk of mortality in patients with chronic obstructive pulmonary disease. *American journal of respiratory and critical care medicine* 2012;186(2):155-61.
3. NICE. Cardiovascular disease: risk assessment and reduction, including lipid modification. *NICE guidelines [CG181]* Published date: July 2014 2014 Last updated: September 2016
4. Gan WQ, Man SFP, Senthilselvan A, et al. Association between chronic obstructive pulmonary disease and systemic inflammation: a systematic review and a meta-analysis. *Thorax* 2004;59(7):574-80.
5. Agusti A, Edwards LD, Rennard SI, et al. Persistent systemic inflammation is associated with poor clinical outcomes in COPD: a novel phenotype. *PloS one* 2012;7(5):e37483.
6. Quist-Paulsen P. Statins and inflammation: an update. *Current Opinion in Cardiology* 2010;25(4)
7. Cao C, Wu Y, Xu Z, et al. The effect of statins on chronic obstructive pulmonary disease exacerbation and mortality: a systematic review and meta-analysis of observational research. *Scientific reports* 2015;5:16461.
8. Ingebrigtsen TS, Marott JL, Nordestgaard BG, et al. Statin use and exacerbations in individuals with chronic obstructive pulmonary disease. *Thorax* 2015;70(1):33-40.
9. Walsh A, Perrem L, Khashan AS, et al. Statins versus placebo for people with chronic obstructive pulmonary disease. *The Cochrane database of systematic reviews* 2019;7(7):CD011959-CD59.
10. Suissa S. Immortal time bias in observational studies of drug effects. *Pharmacoepidemiology and drug safety* 2007;16(3):241-9.
11. Danaei G, Tavakkoli M, Hernan MA. Bias in observational studies of prevalent users: lessons for comparative effectiveness research from a meta-analysis of statins. *American journal of epidemiology* 2012;175(4):250-62.
12. Young RP, Hopkins RJ, Agusti A. Statins as adjunct therapy in COPD: how do we cope after STATCOPE? *Thorax* 2014;69(10):891-4.
13. Herrett E, Gallagher AM, Bhaskaran K, et al. Data Resource Profile: Clinical Practice Research Datalink (CPRD). *Int J Epidemiol* 2015;44(3):827-36.
14. Williams T, van Staa T, Puri S, et al. Recent advances in the utility and use of the General Practice Research Database as an example of a UK Primary Care Data resource. *Ther Adv Drug Saf* 2012;3(2):89-99.
15. Quint JK, Müllerova H, DiSantostefano RL, et al. Validation of chronic obstructive pulmonary disease recording in the Clinical Practice Research Datalink (CPRD-GOLD). 2014;4(7):e005540.
16. Danese MD, Gleeson M, Kutikova L, et al. Management of lipid-lowering therapy in patients with cardiovascular events in the UK: a retrospective cohort study. *BMJ open* 2017;7(5):e013851-e51.

For peer review only - http://bmjopen.bmj.com/site/about/guidelines.xhtml

17. Rothnie KJ, Mullerova H, Hurst JR, et al. Validation of the Recording of Acute Exacerbations of COPD in UK Primary Care Electronic Healthcare Records. *PloS one* 2016;11(3):e0151357.
18. Rothnie KJ, Mullerova H, Thomas SL, et al. Recording of hospitalizations for acute exacerbations of COPD in UK electronic health care records. *Clin Epidemiol* 2016;8:771-82.
19. Mullerova H, Shukla A, Hawkins A, et al. Risk factors for acute exacerbations of COPD in a primary care population: a retrospective observational cohort study. *BMJ open* 2014;4(12):e006171.
20. Calverley PM, Tetzlaff K, Dusser D, et al. Determinants of exacerbation risk in patients with COPD in the TIOSPIR study. *International journal of chronic obstructive pulmonary disease* 2017;12:3391-405.
21. Amorim LD, Cai J. Modelling recurrent events: a tutorial for analysis in epidemiology. *Int J Epidemiol* 2014;44(1):324-33.
22. Neukamm A, Hoiseth AD, Einvik G, et al. Rosuvastatin treatment in stable chronic obstructive pulmonary disease (RODEO): a randomized controlled trial. *Journal of internal medicine* 2015;278(1):59-67.
23. Criner GJ, Connett JE, Aaron SD, et al. Simvastatin for the prevention of exacerbations in moderate-to-severe COPD. *The New England journal of medicine* 2014;370(23):2201-10.
24. Schenk P, Spiel AO, Hüttinger F, et al. Can Simvastatin reduce COPD Exacerbations? A Randomised Double Blind Controlled Study. *The European respiratory journal* 2021
25. Morgan AD, Zakeri R, Quint JK. Defining the relationship between COPD and CVD: what are the implications for clinical practice? *Therapeutic advances in respiratory disease* 2018;12:1753465817750524.
26. Rabe KF, Hurst JR, Suissa S. Cardiovascular disease and COPD: dangerous liaisons? *European respiratory review : an official journal of the European Respiratory Society* 2018;27(149)
27. Collins R, Reith C, Emberson J, et al. Interpretation of the evidence for the efficacy and safety of statin therapy. *Lancet (London, England)* 2016;388(10059):2532-61.
28. He Y, Li X, Gasevic D, et al. Statins and Multiple Noncardiovascular Outcomes: Umbrella Review of Meta-analyses of Observational Studies and Randomized Controlled Trials. *Annals of internal medicine* 2018;169(8):543-53.
29. Chou R, Dana T, Blazina I, et al. Statins for Prevention of Cardiovascular Disease in Adults: Evidence Report and Systematic Review for the US Preventive Services Task Force. *Jama* 2016;316(19):2008-24.
30. Mansournia MA, Etminan M, Danaei G, et al. Handling time varying confounding in observational research. *BMJ (Clinical research ed)* 2017;359:j4587.
31. Ingebrigtsen TS, Marott JL, Nordestgaard BG, et al. Low use and adherence to maintenance medication in chronic obstructive pulmonary disease in the general population. *J Gen Intern Med* 2015;30(1):51-59.
32. Forde I, Chandola T, Raine R, et al. Socioeconomic and ethnic differences in use of lipid-lowering drugs after deregulation of simvastatin in the UK: the Whitehall II prospective cohort study. *Atherosclerosis* 2011;215(1):223-8.
33. Paudyal V, Hansford D, Cunningham S, et al. Pharmacists' perceived integration into practice of over-the-counter simvastatin five years post reclassification. *International journal of clinical pharmacy* 2012;34(5):733-8.

For peer review only

For peer review only - http://bmjopen.bmj.com/site/about/guidelines.xhtml

Figure 1. Selection of the study population

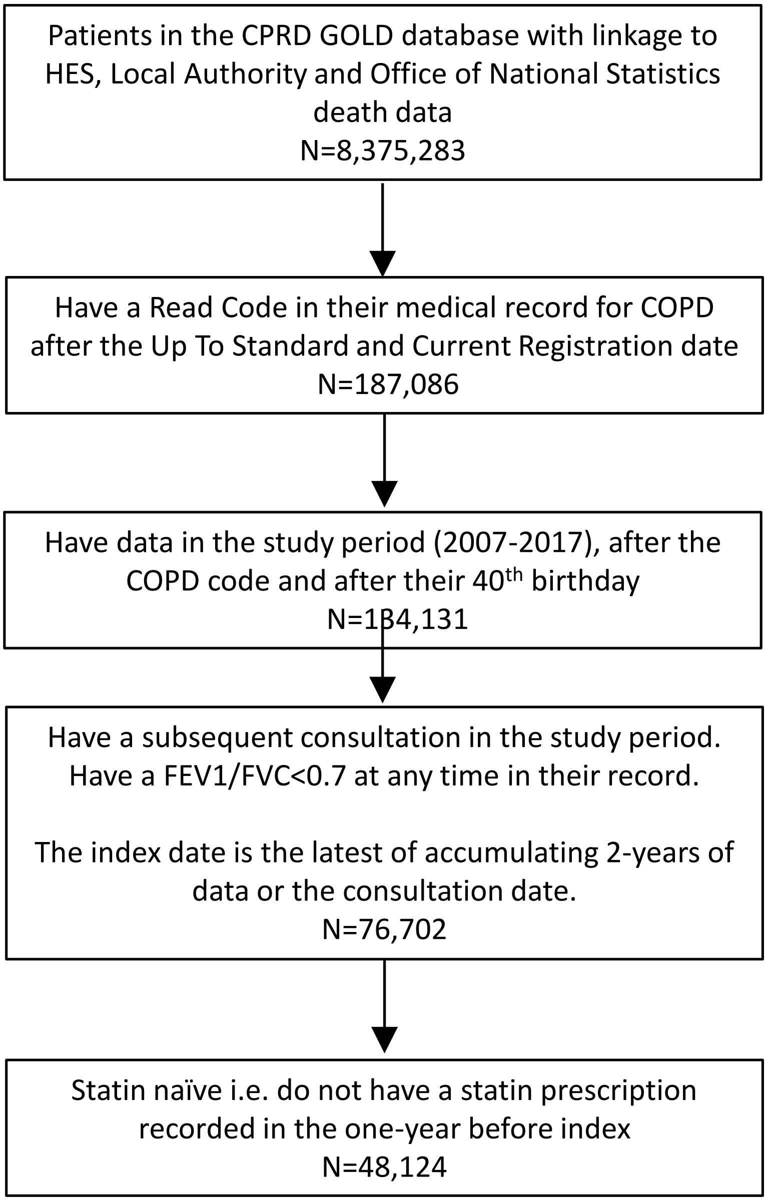

STROBE 2007 (v4) Statement—Checklist of items that should be included in reports of *cohort studies*

Section/Topic	Item #	Recommendation	Reported on page #
**Title and abstract**	1	(*a*) Indicate the study's design with a commonly used term in the title or the abstract	Title & Abstract
		(*b*) Provide in the abstract an informative and balanced summary of what was done and what was found	2-3
**Introduction**			
Background/rationale	2	Explain the scientific background and rationale for the investigation being reported	3-4
Objectives	3	State specific objectives, including any prespecified hypotheses	3
**Methods**			
Study design	4	Present key elements of study design early in the paper	4
Setting	5	Describe the setting, locations, and relevant dates, including periods of recruitment, exposure, follow-up, and data collection	4
Participants	6	(*a*) Give the eligibility criteria, and the sources and methods of selection of participants. Describe methods of follow-up	5
		(*b*) For matched studies, give matching criteria and number of exposed and unexposed	NA
Variables	7	Clearly define all outcomes, exposures, predictors, potential confounders, and effect modifiers. Give diagnostic criteria, if applicable	5
Data sources/ measurement	8*	For each variable of interest, give sources of data and details of methods of assessment (measurement). Describe comparability of assessment methods if there is more than one group	6
Bias	9	Describe any efforts to address potential sources of bias	4
Study size	10	Explain how the study size was arrived at	Fig 1
Quantitative variables	11	Explain how quantitative variables were handled in the analyses. If applicable, describe which groupings were chosen and why	7
Statistical methods	12	(*a*) Describe all statistical methods, including those used to control for confounding	7
		(*b*) Describe any methods used to examine subgroups and interactions	NA
		(*c*) Explain how missing data were addressed	7
		(*d*) If applicable, explain how loss to follow-up was addressed	5
		(*e*) Describe any sensitivity analyses	7
**Results**			

For peer review only

Participants	13*	(a) Report numbers of individuals at each stage of study—eg numbers potentially eligible, examined for eligibility, confirmed eligible, included in the study, completing follow-up, and analysed	Fig 1, p7
		(b) Give reasons for non-participation at each stage	Fig 1
		(c) Consider use of a flow diagram	Fig 1
Descriptive data	14*	(a) Give characteristics of study participants (eg demographic, clinical, social) and information on exposures and potential confounders	Table 1
		(b) Indicate number of participants with missing data for each variable of interest	Table 1
		(c) Summarise follow-up time (eg, average and total amount)	10
Outcome data	15*	Report numbers of outcome events or summary measures over time	10, Table 2
Main results	16	(*a*) Give unadjusted estimates and, if applicable, confounder-adjusted estimates and their precision (eg, 95% confidence interval). Make clear which confounders were adjusted for and why they were included	Table 2
		(*b*) Report category boundaries when continuous variables were categorized	P7, Table 2
		(*c*) If relevant, consider translating estimates of relative risk into absolute risk for a meaningful time period	NA
Other analyses	17	Report other analyses done—eg analyses of subgroups and interactions, and sensitivity analyses	Table2, Table 3
**Discussion**			
Key results	18	Summarise key results with reference to study objectives	P13
**Limitations**			
Interpretation	20	Give a cautious overall interpretation of results considering objectives, limitations, multiplicity of analyses, results from similar studies, and other relevant evidence	P13-p15
Generalisability	21	Discuss the generalisability (external validity) of the study results	P13-P15
**Other information**			
Funding	22	Give the source of funding and the role of the funders for the present study and, if applicable, for the original study on which the present article is based	P16

*Give information separately for cases and controls in case-control studies and, if applicable, for exposed and unexposed groups in cohort and cross-sectional studies.

Note: An Explanation and Elaboration article discusses each checklist item and gives methodological background and published examples of transparent reporting. The STROBE checklist is best used in conjunction with this article (freely available on the Web sites of PLoS Medicine at http://www.plosmedicine.org/, Annals of Internal Medicine at http://www.annals.org/, and Epidemiology at http://www.epidem.com/). Information on the STROBE Initiative is available at www.strobe-statement.org.

For peer review only

BMJ Open

Statin prescription in patients with chronic obstructive pulmonary disease and risk of exacerbations: a retrospective cohort study in the Clinical Practice Research Datalink

Journal:	*BMJ Open*
Manuscript ID	bmjopen-2021-050757.R2
Article Type:	Original research
Date Submitted by the Author:	01-Nov-2021
Complete List of Authors:	Smith, Margaret; University of Oxford, Nuffield Department of Primary Care Health Sciences; Oxford University Hospitals NHS Foundation Trust, NIHR Oxford Biomedical Research Centre<br>Ashdown, Helen; University of Oxford, Nuffield Department of Primary Care Health Sciences<br>Sheppard, James; University of Oxford, Nuffield Department of Primary Care Health Sciences<br>Butler, Christopher C.; University of Oxford, Nuffield Department of Primary Health Care Sciences<br>Bankhead, Clare; University of Oxford, Nuffield Department of Primary Care Health Sciences; Oxford University Hospitals NHS Foundation Trust, NIHR Oxford Biomedical Research Centre
**Primary Subject Heading**:	Respiratory medicine
Secondary Subject Heading:	General practice / Family practice, Pharmacology and therapeutics
Keywords:	Chronic airways disease < THORACIC MEDICINE, Adult thoracic medicine < THORACIC MEDICINE, THERAPEUTICS

SCHOLARONE™
Manuscripts

I, the Submitting Author has the right to grant and does grant on behalf of all authors of the Work (as defined in the below author licence), an exclusive licence and/or a non-exclusive licence for contributions from authors who are: i) UK Crown employees; ii) where BMJ has agreed a CC-BY licence shall apply, and/or iii) in accordance with the terms applicable for US Federal Government officers or employees acting as part of their official duties; on a worldwide, perpetual, irrevocable, royalty-free basis to BMJ Publishing Group Ltd ("BMJ") its licensees and where the relevant Journal is co-owned by BMJ to the co-owners of the Journal, to publish the Work in this journal and any other BMJ products and to exploit all rights, as set out in our licence.

The Submitting Author accepts and understands that any supply made under these terms is made by BMJ to the Submitting Author unless you are acting as an employee on behalf of your employer or a postgraduate student of an affiliated institution which is paying any applicable article publishing charge ("APC") for Open Access articles. Where the Submitting Author wishes to make the Work available on an Open Access basis (and intends to pay the relevant APC), the terms of reuse of such Open Access shall be governed by a Creative Commons licence – details of these licences and which Creative Commons licence will apply to this Work are set out in our licence referred to above.

Other than as permitted in any relevant BMJ Author's Self Archiving Policies, I confirm this Work has not been accepted for publication elsewhere, is not being considered for publication elsewhere and does not duplicate material already published. I confirm all authors consent to publication of this Work and authorise the granting of this licence.

For peer review only

Statin prescription in patients with chronic obstructive pulmonary disease and risk of exacerbations: a retrospective cohort study in the Clinical Practice Research Datalink

Dr Margaret C Smith[1,2], Dr Helen Frances Ashdown[1], Dr James Peter Sheppard[1,2], Professor Christopher C Butler[1], Professor Clare Bankhead[1,2]

[1] Nuffield Department of Primary Care Health Sciences, University of Oxford, Oxford, UK

[2] NIHR Oxford Biomedical Research Centre, Oxford University Hospitals NHS Foundation Trust

Corresponding Author: Dr Margaret Smith,

Nuffield Department of Primary Care Health Sciences, University of Oxford, Radcliffe Primary Care Building, Radcliffe Observatory Quarter, Woodstock Road, Oxford, OX2 6GG.

Margaret.Smith@phc.ox.ac.uk

Word count of main text 3816:

Number of Tables: 3; Number of Figures: 1

Key words: chronic obstructive pulmonary disease, exacerbation, statin, death, urinary tract infection, cohort study

For peer review only

ABSTRACT

Objective

Observational studies have suggested a beneficial effect of taking statins on frequency of chronic obstructive pulmonary disease (COPD) exacerbations. However, clinical trials of statins in people with COPD did not confirm those results. This study aimed to investigate this association using a methodological approach, which reduces the biases associated with some previous observational study designs.

Design

Retrospective cohort study comparing new-users of statins with non-users.

Setting

General practices in England contributing to the Clinical Practice Research Datalink in 2007-2017, with linkage to data on Hospital Episode Statistics inpatient episodes.

Participants

48,124 people with COPD, aged over 40 years, who had not been prescribed statin in the previous year.

Exposure

Participants became new-users of statins at their first prescription for a statin during follow-up. They were then assumed to remain statin users. Statin users were compared with non-users.

Outcomes

Primary outcomes were COPD exacerbation, or severe exacerbation requiring hospitalisation. Secondary outcomes were death from any cause (for comparison with other studies) and urinary tract infection (negative-control). Maximum follow-up was 3 years. Adjusted hazard ratios (HR) were calculated using time-dependent Cox regression. The Andersen-Gill model was used for recurrent exacerbations. Covariates included demographic variables, variables related to COPD severity, cardiovascular comorbidities as time-dependent variables, and other comorbidities at baseline.

For peer review only

Results

7,266 participants became new-users of statins over an average 2.5 years of follow-up. In total 30,961 people developed an exacerbation, 8,110 severe exacerbation, 3,650 urinary tract infection and 5,355 died. Adjusted HR (95% CI) in statin users compared to non-users were first exacerbation 1.01 (0.97-1.06), severe exacerbation 0.92 (0.85-1.00), number of exacerbations 1.00 (0.97-1.04), urinary tract infection 1.10 (0.98-1.24) and death 0.63 (0.57-0.70).

Conclusions

In this study of health records from a Primary Care database statin use in people with COPD was not associated with a lower risk of COPD exacerbation.

(300 words)

STRENGTHS AND LIMITATIONS OF THIS STUDY

- We used a large real-world cohort of over 48,000 people with COPD who were statin naïve at study entry.
- The exposure was defined as new statin prescription in follow-up and analysed as a time-dependent variable. New user designs are less susceptible to prevalent-user bias.
- We included secondary outcomes and various levels of confounding adjustment to investigate some potential sources of bias.
- This study identified statin users from their first prescripton of statins. We cannot be certain that the medication was taken and adherence may have declined over follow-up.
- As this was an observational study, we cannot be certain that residual confounding or other biases did not affect our results

INTRODUCTION

Chronic obstructive pulmonary disease (COPD) is characterised by progressive and irreversible airflow limitation, together with sudden flare-ups or exacerbations. Frequent exacerbations accelerate loss of lung function and are associated with poorer quality of life and poor survival.[1] COPD is often associated with the presence of other co-morbidities, of which cardiovascular disease (CVD) is one of the most significant.[2] Therefore many people with COPD are likely to be

For peer review only - http://bmjopen.bmj.com/site/about/guidelines.xhtml

For peer review only

recommended medication for primary or secondary prevention of CVD, including with statins.[3] Increased levels of biomarkers reflecting systemic inflammation are also part of the pathology of COPD. Levels of inflammatory biomarkers increase during the acute phase of an exacerbation, and in individuals with stable COPD elevated levels of these biomarkers are associated with higher frequency of exacerbations.[4 5] Statins have been found to have anti-inflammatory effects, as well as reducing cholesterol.[6] Therefore there is a theoretical possibility that statins may have a beneficial role in COPD management over and above reducing cardiovascular risk.

Several observational studies have found an association between statin use and fewer exacerbations in COPD patients.[7 8] However randomised controlled trials have failed to confirm any such associations.[9] Several explanations for the conflict of trial results with those from observational studies have been proposed. Firstly, immortal time bias arises from misspecification of exposures so that there is a span of cohort follow-up during which, because of exposure definition, the outcome under study cannot occur.[10] Secondly, prevalent user bias occurs when risk of an outcome changes over time in a population exposed to a drug treatment.[11] Lastly, the population and treatment in the clinical trials were very precisely defined and unlikely to be typical of the general population.[12]

A study design in which all patients are statin naïve at study entry, and in which initiation of statins is included as a time dependent variable can reduce the likelihood of some of these biases. This study aimed to use such a study design to examine the association between statin prescription and incidence of COPD exacerbations in a general population of patients attending routine primary care.

METHODS

Study design

The study is a retrospective cohort study comparing new-users of statins with non-users. It was conducted within the Clinical Practice Research Datalink (CPRD) GOLD database, which is a collection of anonymised primary care records from ~700 UK practices. These data have been shown to be representative of the general UK population in terms of age, sex and ethnicity.[13] We used data from the three-quarters of CPRD English practices with linkage to the English Hospital Episode Statistics (HES), and mortality data from the UK Office for National Statistics (ONS), and patient-level social deprivation indices.

For peer review only - http://bmjopen.bmj.com/site/about/guidelines.xhtml

Patient and Public Involvement

As this is a retrospective database study there are limited opportunities for Patient and Public Involvement in study design or conduct. During the development of the protocol, we discussed experience of exacerbations with three people who had COPD themselves or had experience of caring for people with COPD.

Population and exposure

We included patients aged at least 40 years, who consulted between Jan 2007 and July 2017, and had a preceding Read code for COPD in their medical records within research-quality data. Research quality data fulfils basic CPRD-defined quality criteria at the practice (recorded death rates are within a pre-defined range based on the UK average and that there is continuity in recording of deaths and other events) and patient level (included patients are permanently registered at their practice with a valid birth year and registration date) .[14] To confirm the presence of COPD, a forced expiratory volume in one second/forced vital capacity (FEV1/FVC) of <0.7 had to also be recorded.[15] To ensure that patients were actively consulting and that there were enough data to identify baseline values of covariates, the index date was defined as the latest of the consultation date and the date of accumulating two years of research quality data in the current practice. Patients in the selected cohort were also all statin naïve as they were only included in the cohort if there had been no statin prescription in the 12 months before the index date. They were defined as newly prescribed users of statins once a prescription was issued on the index date or during follow-up.

Outcomes

Patients were followed-up until they were censored after 3-years, or until the end of the study period or they died or left their practice or their practice stopped contributing data to CPRD GOLD, if any of these occurred within 3-years. Follow-up was limited to 3 years because we expected adherence to statins to drop-off over time.[16]

Our primary outcomes were any COPD exacerbation and severe exacerbation requiring hospitalisation. An acute exacerbation of COPD in the Primary Care record was defined as a code for acute exacerbation, code for lower respiratory tract infection, prescriptions for 5-14 days of exacerbation-specific antibiotics and oral steroid on the same date, or a symptom of exacerbation together with a prescription for exacerbation-specific antibiotics or oral steroids.[17] Exacerbations were also identified from the HES inpatient record, from a COPD or acute respiratory codes as the primary diagnosis of hospitalisation (ICD-10 J00, J06, J09-18, J20-22, J40-44, J96), or from a code for acute exacerbation of COPD anywhere in the hospitalisation record (J44.0, J44.1).[18] We identified all

For peer review only - http://bmjopen.bmj.com/site/about/guidelines.xhtml

exacerbations in follow-up. To prevent double counting, events indicating exacerbations within 14 days of each other were assumed to describe the same exacerbation, with the exacerbation date taken from the first code. A severe exacerbation was any exacerbation with a relevant code, as described above, in the HES inpatient data at some point in its duration.

Secondary outcomes were death from any cause, and urinary tract infection (UTI). Death was chosen so that we could compare the observed association with results from randomised controlled trials in the general population. UTI recorded in Primary Care data was included as a negative-control outcome because we did not expect any association with statin use. A strong association between statin use and UTI might indicate some bias in the study design.

Covariates

We assessed potential confounders at the index date and time dependently. For the purpose of adjustment in regression analyses covariates were combined into groups: lifestyle and demographic factors, markers of COPD severity, CVD-related variables, and other co-morbidities or health-related variables.

- Lifestyle and demographic variables (basic adjustment) included gender, age at index, deprivation (as quintile of Index of Multiple Deprivation), and smoking status before index.
- Covariates related to COPD severity were FEV_1 percent of predicted, number of COPD exacerbations, severe exacerbation requiring hospitalisation, regular prescription of COPD-related medication (short-acting bronchodilator, long-acting bronchodilator, inhaled corticosteroid, nebuliser), and receiving oxygen therapy.[19 20] The most recent FEV_1 percent of predicted in the two years prior to index was extracted. All other variables were assessed in the one-year before index. COPD exacerbations were defined as for outcomes. Regular prescription was defined as at least three prescriptions in the year.
- CVD-related variables were previous CVD, diabetes and hypertension. These variables were assessed as if they had occurred ever before index and time-dependently during follow-up. We included this group of variables as time-dependent covariates in main analyses because they are very likely to be associated with statin exposure. CVD was defined as a diagnosis code for one of coronary heart disease, stroke, heart failure or peripheral artery disease in the Primary Care data or in the linked HES inpatient data. Hypertension was defined as a diagnosis code or a prescription for an antihypertensive medication. We also included body mass index (BMI) (the most recent measurement in two years before index) in this group of covariates.

For peer review only - http://bmjopen.bmj.com/site/about/guidelines.xhtml

- Comorbidities and other health-related variables including asthma, other respiratory disease (bronchiectasis, cystic fibrosis, alpha-1 anti-trypsin-deficiency, and interstitial lung disease), lung cancer, rheumatoid arthritis, gastro-oesophageal reflux disease and osteoporosis were assessed from codes in Primary Care and HES inpatient data recorded at any time before the index date. Depression, influenza vaccination, and number of doctor or nurse consultations were assessed in Primary Care data in the year before index.

Statistical Analysis

We used Cox regression to investigate the association of statin use with time to first event for exacerbation, severe exacerbation, death or UTI. The proportional hazards assumption was assessed for the time-independent covariates using graphical methods (-ln –ln of survival curves). For analyses of recurrent exacerbation, we used the Andersen and Gill model.[21] Statin use was defined as a binary time-dependent variable. All patients were non-users of statin immediately before the index date. They became new-users of statins at their first prescription of statin on the index date or during follow-up. In main analyses, we took an intention to treat approach in which people were assumed to remain statin users from the initial prescription until they were censored after a maximum of 3-years of follow-up. We censored after 3-years because we expected adherence to statins to fall over time, which could dilute the observed effect size. The analyses were repeated with combinations of the different sets of covariates to try to understand the role of confounding. All analyses were conducted in Stata 16 (StataCorp, Texas, US).

Age at index and BMI were included in analyses as continuous covariates. FEV_1 percent of predicted was included as a categorical variable (≥80%, 50-<80%, 30-<50%, <30%). Quintile of IMD, number of exacerbations (0, 1, 2, 3, 4, ≥5) and smoking status (never, ex, current) were also included as categorical variables. Other covariates were included as binary variables. Time dependent variables were incorporated by splitting patient records at the date of the first prescription for statin or when CVD, diabetes or hypertension were first recorded.

Missing values of BMI or FEV_1 percent of predicted were imputed with multiple imputation by chained equations, using a linear regression model for imputing FEV_1 percent of predicted and predictive mean matching for BMI, and including all variables to be included in planned analyses (including outcomes). Thirty imputed data sets were calculated and estimates from regressions were pooled using Rubin's rules. People with missing smoking data were assumed non-smokers. Missing values of other variables were interpreted as a null value e.g. no prescription or no comorbidity. We

For peer review only - http://bmjopen.bmj.com/site/about/guidelines.xhtml

also repeated analyses restricted to people with complete data. We did not find any substantive difference in results (data not shown).

In secondary analyses, we censored 90-days after the last recorded statin prescription to investigate the effect of non-adherence on associations. In main analyses that were adjusted for CVD-related variables we adjusted for these variables at index and time-dependently. A *post hoc* sensitivity analysis was also conducted for the death outcome in which we adjusted for CVD variables assessed at baseline rather than the time-dependent CVD variables.

RESULTS

Baseline characteristics

A total of 8,375,283 people were available with complete linkage within the CPRD GOLD database (Figure 1). Within these, we identified 76,702 people aged 40+ years who fulfilled the definition of a COPD diagnosis, had a recent record of consulting their GP and had data in the study period. Of these, 48,124 were statin naïve and were included in the cohort. Of these, 7,266 had at least one statin prescription on the index date or subsequently during a maximum of 3-years of follow-up. For 40,858 there was no record of a statin prescription in follow-up.

People who subsequently started on statins i.e. became new-users of statins, had different baseline characteristics compared to those who did not (Table 1). They were less likely to be female (42% compared to 50%). They were also more likely to have social deprivation above the median (57% vs. 54%) but slightly less likely to be never smokers (8% vs. 10%). Variables related to COPD severity indicated that COPD was on average slightly less severe at baseline in the new statin users. Those who became statin users had a slightly higher body mass index at baseline (27 vs 26 kgm^{-2}) and considerably higher prevalence of hypertension (50% vs 39%), CVD (14% vs 8%) and diabetes (6% vs 2%) at baseline. More had hypertension, CVD or diabetes newly recorded in follow-up. New statin-users were also more like to have an influenza vaccination in the year before baseline (72% vs 66%), and more likely to make 5+ visits to a GP (57% vs 55%) or nurse (24% vs 22%).

For peer review only - http://bmjopen.bmj.com/site/about/guidelines.xhtml

Table 1. Descriptive statistics for the study population according to whether they received a statin prescription in follow-up i.e. became new-users of statins. Baseline characteristics are given for all variables. New cases over follow-up are also given for cardiovascular disease and related conditions. Statistics are n (%) unless otherwise stated.

	Prescription for statin in follow-up	
**Variable**	**No (N=40858)**	**Yes (N=7266)**
Index date (median)	March 2008	July 2007
**Lifestyle and demographic**		
Female	20375 (50%)	3045 (42%)
Mean(SD) age (years)	67(12)	68(10)
Deprivation above median (IMD 2007)	22109 (54%)	4174 (57%)
Smoking status		
Never	4153 (10%)	604 (8%)
Ex	19978 (49%)	3661 (50%)
Current	16524 (40%)	2973 (41%)
Missing	203 (0%)	28 (0%)
**Related to COPD severity**		
Mean(SD) FEV_1 (percent of predicted)	63(21)	65(21)
Missing	11278 (28%)	2062 (28%)
FEV_1 percent of predicted		
≥ 80	6073 (15%)	1123 (15%)
≥ 50 - < 80	15152 (37%)	2788 (38%)
≥ 30 - < 50	6966 (17%)	1100 (15%)
< 30	1389 (3%)	193 (3%)
Missing	11278 (28%)	2062 (28%)
Number of COPD exacerbations in 1-year before index		
0	20336 (50%)	3785 (52%)
1	11154 (27%)	1914 (26%)
2	5073 (12%)	884 (12%)
3	2237 (5%)	346 (5%)
4	1024 (3%)	175 (2%)
≥5	1034 (3%)	162 (2%)
At least one severe exacerbation in 1-year before index	2981 (7%)	499 (7%)
Regular prescription (3+) of COPD medication in 1-year before index		
Short acting bronchodilator	20936 (51%)	3661 (50%)
Long acting bronchodilator	18068 (44%)	3075 (42%)

For peer review only - http://bmjopen.bmj.com/site/about/guidelines.xhtml

Inhaled corticosteroid	19136 (47%)	3323 (46%)
Nebuliser	1654 (4%)	230 (3%)
On oxygen therapy	423 (1%)	49 (1%)
**Related to cardiovascular disease**		
Diabetes		
Recorded before index date	1007 (2%)	462 (6%)
New over follow-up	482 (1%)	693 (10%)
Hypertension		
Recorded before index date	16093 (39%)	3661 (50%)
New over follow-up	4632 (11%)	2066 (28%)
Cardiovascular disease		
Recorded before index date	3241 (8%)	991 (14%)
New over follow-up	1812 (4%)	1984 (27%)
Body mass index (kg m^{-2}) Mean(SD)	26(6)	27(6)
Missing	10838 (27%)	1679 (23%)
**Comorbidities and other health-related recorded before index date**		
Respiratory comorbidities		
Asthma	10818 (26%)	1890 (26%)
Other respiratory disease	1289 (3%)	195 (3%)
Other comorbidities		
Lung cancer	331 (1%)	46 (1%)
Depression in 1-year before index	2808 (7%)	474 (7%)
Rheumatoid arthritis	700 (2%)	123 (2%)
Gastro-oesophageal reflux disease	4966 (12%)	995 (14%)
Osteoporosis	2113 (5%)	300 (4%)
Influenza vaccination in 1-year before index	27143 (66%)	5211 (72%)
Regular (5+) contacts with practice staff in 1-year before index		
GP	22525 (55%)	4153 (57%)
Nurse	8979 (22%)	1772 (24%)

SD, standard deviation; IMD, index of multiple deprivation, COPD chronic obstructive pulmonary disease; FEV_1, forced expiratory volume in one second percent of predicted; GP, general practitioner

Associations of statin use with outcomes

The mean follow-up was 2.46 years. 17,350 (36%) people were censored before the end of the maximum 3 years of follow-up, due to death (5,355 (11%)), end of the study period (1,973 (4%)), or

For peer review only - http://bmjopen.bmj.com/site/about/guidelines.xhtml

For peer review only

leaving their current GP practice or the end of downloaded data in CPRD from their practice (10,022 (21%)). Over follow-up, there were 106,961 exacerbations recorded. 30,961 (64%) people had at least one exacerbation, 8,110 (17%) were hospitalised for a severe exacerbation and 3,650 (8%) had at least one UTI diagnosed. The mean follow-up in nonusers of statins (to censoring, death or starting on statins) was 2.23 years (total 107039 years). The mean follow-up after starting to use statins was 1.56 years (total 11307 person years.

The first statin prescription was simvastatin in 6,089 (84%) of the new users. It was atorvastatin for 989 (14%), pravastatin for 133 (2%), rosuvastatin for 53 (1%) and fluvastatin for two people. The number of statin prescriptions per person was 9 (4-18) (median and interquartile range). Amongst those whose first prescription was for simvastatin, 397(7%) had a prescription for a daily dose of 10mg, 1822 (30%) for 20mg, 3864 (64%) for 40mg and 6 (<1%) for 80mg.

COPD exacerbation

There was no association between statins and COPD exacerbation after adjustment for basic demographic variables (hazard ratio (HR) 0.99, 95%CI 0.94-1.03) (Table 2). Additionally adjusting for cardiovascular risk factors resulted in a small statistically significant beneficial effect of statins (HR 0.94, 95%CI 0.90-0.99) (Table 2). The association was constant after further adjustment for variables related to severity of COPD (HR 1.01, 95%CI 0.96-1.06). This remained the case after adjustment for all covariates (HR 1.01, 95%CI 0.96-1.06) and in analyses censoring 90-days after the last statin prescription (Table 3). A similar pattern of HR with different adjustments was seen for the recurrent COPD exacerbation outcome and for severe COPD exacerbations. The HR for recurrent COPD exacerbations adjusted for all covariates was 1.00 (95%CI 0.97-1.04). The HR for severe exacerbations was marginally significant after adjustment for all covariates (HR 0.92, 95% CI 0.84 – 0.99).

Table 2. Hazard ratios (HR) for association of chronic obstructive pulmonary disease (COPD) exacerbation, urinary tract infection or death with statin use

Outcome	Covariates in model*	HR	(95% CI)	P
Primary outcomes				
COPD exacerbation (N events in statin users, non-users= 1893, 29068)				
	Unadjusted	0.99	(0.94 - 1.04)	0.652
	Basic	0.99	(0.94 - 1.03)	0.583
	Basic+CVD	0.94	(0.90 - 0.99)	0.014
	Basic+COPD	1.03	(0.99 - 1.08)	0.168
	Basic+COPD+CVD	1.01	(0.96 - 1.06)	0.736

For peer review only - http://bmjopen.bmj.com/site/about/guidelines.xhtml

	All covariates	1.01	(0.96 - 1.06)	0.657

	All covariates	1.01	(0.96 - 1.06)	0.657
COPD exacerbation, recurrent events (N events in statin users, non-users== 10352, 96609)				
	Unadjusted	1.02	(0.98 - 1.06)	0.359
	Basic	1.02	(0.98 - 1.06)	0.334
	Basic+CVD	0.94	(0.91 - 0.98)	0.002
	Basic+COPD	1.04	(1.01 - 1.08)	0.010
	Basic+COPD+CVD	1.00	(0.97 - 1.03)	0.999
	All covariates	1.00	(0.97 - 1.04)	0.885
Severe COPD exacerbation (N events in statin users, non-users= = 722, 7388)				
	Unadjusted	1.05	(0.97 - 1.14)	0.217
	Basic	1.00	(0.92 - 1.08)	0.977
	Basic+CVD	0.80	(0.74 - 0.87)	<0.001
	Basic+COPD	1.07	(0.99 - 1.16)	0.087
	Basic+COPD+CVD	0.90	(0.83 - 0.98)	0.016
	All covariates	0.92	(0.84 – 0.99)	0.034
Secondary outcomes				
Death from any cause (N events in statin users, non-users= = 466, 4889)				
	Unadjusted	0.87	(0.79 - 0.96)	0.005
	Basic	0.85	(0.77 - 0.93)	0.001
	Basic+CVD	0.58	(0.53 - 0.64)	<0.001
	Basic+COPD	0.88	(0.80 - 0.97)	0.009
	Basic+COPD+CVD	0.63	(0.57 - 0.69)	<0.001
	All covariates	0.63	(0.57 - 0.70)	<0.001
	Basic+COPD+Baseline CVD	0.83	(0.76 - 0.92)	<0.001
Urinary tract infection (N events in statin users, non-users= 360, 3290)				
	Unadjusted	1.19	(1.07 - 1.33)	0.002
	Basic	1.28	(1.14 - 1.43)	<0.001
	Basic+CVD	1.07	(0.95 - 1.20)	0.273
	Basic+COPD	1.29	(1.16 - 1.44)	<0.001
	Basic+COPD+CVD	1.09	(0.97 - 1.22)	0.159
	All covariates	1.10	(0.98 - 1.23)	0.112

* Covariates included in the models. Basic adjustment, lifestyle and demographic variables including gender, age, IMD quintile (categorical), smoking status (never, ex, current); COPD, severity of chronic obstructive disease (COPD) at the index date including exacerbation frequency (categorised as 0,1,2,3,4,5+), severe exacerbation requiring hospitalisation, forced expiratory volume in one-second percent of predicted (categorised as 80, 50-<80, 30-<50, <30) , regular prescriptions for COPD-related medications, received oxygen; CVD, cardiovascular disease (CVD) related variables at index and as time-dependent variables including diabetes, hypertension, CVD diagnosis, body mass index (at index); All covariates, included Basic+COPD+CVD groups of covariates and also other comorbidities and health-related covariates recorded at index (see list in Methods). Baseline CVD,

For peer review only - http://bmjopen.bmj.com/site/about/guidelines.xhtml

CVD related variables at the index date (post hoc sensitivity analysis conducted for the death outcome only).

Table 3. Hazard ratios (HR) for association of chronic obstructive pulmonary disease (COPD) exacerbation, death or urinary tract infection with statin use. Follow-up was censored 90-days after the last statin prescription. HR are adjusted for all covariates*.

Outcome	N events	HR	(95% CI)	P
Primary outcomes				
COPD exacerbation	17,633	1.02	(0.94-1.09)	0.688
COPD exacerbation, recurrent events	102,342	1.00	(0.96-1.04)	0.995
Secondary outcomes				
Severe COPD exacerbation	4,365	0.89	(0.78-1.01)	0.080
Death	2,828	0.50	(0.42-0.60)	<0.001
Urinary tract infection	2,054	0.99	(0.82-1.20)	0.943

* Covariates included in the models. Variables included in basic adjustment; variables related to severity of chronic obstructive disease (COPD) at the index date; cardiovascular disease related variables at index and as time-dependent variables; other comorbidities and health-related covariates recorded at index. See Methods and legend to Table 2 for details.

Secondary outcomes

Taking statins was associated with a lower risk of death from any cause. The HR after basic adjustment was 0.85 (95%CI 0.77 - 0.93) (Table 2). The association was considerably strengthened after adjusting for baseline and time-dependent CVD risk factors. After adjustment for all covariates the HR was 0.63 (95% CI 0.57 - 0.70). In the post hoc sensitivity analysis in which adjustment was for all covariates but with CVD restricted to baseline values the HR was 0.83 (95% CI 0.76 - 0.92). There was an increased risk of UTI in people taking statins after basic adjustment (HR 1.28, 95%CI 1.14 - 1.43). This was reduced and no longer statistically significant after adjusting for all covariates (HR 1.10, 95%CI 0.98 - 1.23).

DISCUSSION

In this study of people with COPD who were followed up for a maximum of 3-years, we did not find evidence to support a beneficial effect of statin use on incidence of COPD exacerbations. The hazard ratios were close to one whether we considered the first exacerbation or multiple exacerbations

For peer review only - http://bmjopen.bmj.com/site/about/guidelines.xhtml

after study entry. Using statins was associated with a slightly lower (8%, 95% CI 1%-16%) risk of severe exacerbation requiring hospitalisation after adjusting for all covariates.

Our findings for the primary outcome of COPD exacerbation are consistent with those from most randomised controlled trials[9 22 23], but contrast with those of previous observational studies which have found 30-40% lower risk of exacerbation with taking statins.[7 8] STATCOPE, the larger trial (885 participants) of simvastatin versus placebo for prevention of exacerbations among patients with moderate-severe COPD but no other indications for statin use was stopped early due to lack of benefit of statins on exacerbation rate.[23] The much smaller RODEO trial of rosuvastatin in patients with verified COPD but not CVD also failed to find any benefit of statin on exacerbation rate.[22] Recently published results from another small trial do however indicate some benefit of statin treatment on COPD exacerbation and mortality.[24] An explanation put forward by the authors for these results is that the study population was nearer “real world” than in previous trials as it included patients with subclinical cardiovascular disease. CVD is strongly linked to COPD, [25 26] and so some hospital admissions for severe exacerbation may have been attributable to CVD. The beneficial effect of statins may have arisen as a knock-on effect on severity of COPD exacerbations via reduced incidence of CVD. Observational studies and randomised controlled trials have found possible beneficial effects of statin use on development of many other diseases but as with statins and COPD exacerbations, most of these findings need further rigorous investigation.[27 28]

We included UTI as a negative-control outcome. In other words, we would not expect an association with statin use if confounding was controlled for and if the study was free from other biases. We found that the rate of UTI was slightly increased in statin users, but this association became non-significant after adjustment for cardiovascular risk factors.

Taking statins was associated with a 15% (95% CI 7%-23%) lower risk of death after basic adjustment for demographic variables and a 37% lower risk of death (95% CI 30%-43%) after adjusting for all confounders. Our observed effect size is somewhat larger than has been estimated in trials conducted in the general population, which is about 10%.[27 29] Healthy user biases, or the reverse in which COPD patients not on statins have poorly treated comorbid CVD which contributes to worse outcomes may have contributed to our observation.[12] Very ill patients with a higher risk of death or COPD exacerbation may not have been prescribed statins: patients in our study population who were prescribed statins had on average slightly milder COPD, and patients with end-stage COPD being managed palliatively are unlikely to be initiating a statin.

Those initiating statins had much higher prevalence of CVD-related variables at index date and incident over follow-up so we adjusted for time-dependent CVD. However, we may have

For peer review only - http://bmjopen.bmj.com/site/about/guidelines.xhtml

inadvertently biased some associations by including new CVD diagnoses after statin initiation, because CVD risk is also affected by statin exposure.[30] Therefore time-dependent CVD might also be a mediator on the path between statin use and outcomes. This raises the possibility that all HR that were adjusted for time-dependent CVD variables may have been subject to some degree of over-adjustment. The post hoc sensitivity analysis of the death outcome adjusting for baseline CVD but not time-dependent CVD resulted in an estimate of 17% (95% CI 8%-24%) lower risk of death, which is closer to expectations. However, this estimate is also likely to be biased because it does not allow for confounding by CVD developed after the index date.

Strengths and limitations of this study

Strengths of our study are the use of a large statin-naïve cohort with statin exposure included as a time-dependent exposure to minimise immortal time bias and prevalent user bias; adjustment for a wide range of potential confounding variables; and inclusion of control outcomes to investigate residual confounding.

The CPRD GOLD database is based on Primary Care data and therefore our study population is more generalizable to real-world clinical practice than selected populations recruited for clinical trials. CPRD GOLD has been used previously for a wide range of studies, and has been shown to be representative of the UK population and many variables extracted from the data have been validated externally including those related to COPD.[15 17 18]

Studies using databases of electronic health records also have their limitations. Non-adherence to prescribed statin medications or use of over-the-counter statins could potentially have reduced the size of any observed effect of statins on outcomes. Our assessment of statin use was based on statin prescriptions made in Primary Care. We cannot be certain that medications were dispensed or that patients were taking their medication. One study of individuals with COPD found that the proportion of prescriptions for COPD maintenance medications that were picked up was quite low, as was adherence to these medications.[31] Furthermore low-dose statins have been available over-the-counter in the United Kingdom since 2004, and these would not be recorded in Primary Care records. Over-the-counter statin medication is limited to 10mg simvastatin but most Primary Care prescriptions for statins in our study were for 40mg simvastatin. In addition, the support for over-the-counter statins by pharmacists and clinicians has been limited, and the take-up has been very low.[32 33]

COPD exacerbations are recorded in a number of different ways in Primary Care records and are therefore not straightforward to identify. We used a validated algorithm to identify exacerbations,

For peer review only - http://bmjopen.bmj.com/site/about/guidelines.xhtml

but there is likely to have been some misclassification.[17] Non-differential misclassification is expected to move effect sizes towards the null. We also learnt from discussions with PPI volunteers that some exacerbations might be treated in accident and emergency departments. However, even though these data are available it may be difficult to identify exacerbations in them, and no methodology has been validated yet, so by not including accident and emergency data we may have missed COPD exacerbations, although documentation should be sent to the patient's GP. Missing data is potentially an issue in electronic health record databases: in particular, we did not have an estimate of FEV_1 percent predicted for 28% of included patients.

Other limitations of this study relate to the use of an observational study design. People starting on statins are likely to be different from those not doing so. We included a wide range of confounding variables in our models, nevertheless, we cannot be certain that there is not still some residual confounding. As discussed above, we included variables related to CVD as time-dependent covariates, which may have led to some degree of over-adjustment. We included secondary outcomes to help to identify potential sources of bias, but we cannot be certain that our results are not affected by other sources of bias that we have not considered.

CONCLUSION

We did not find evidence to support an association between statin prescription and reduced risk of exacerbation in people with COPD. These data suggest that in people with COPD and no other indication, statins should not be prescribed in routine primary care to reduce COPD exacerbations. Our findings are consistent with the well-established benefit of statins for reducing death in patients with CVD risk factors, and confirm this in a COPD population.

FIGURE LEGEND

Figure 1 Flow chart describing the selection of the study population from the Clinical Practice Research Datalink (CPRD) GOLD database. FEV1/FVC is the ratio of forced expiratory volume in one second to forced vital capacity.

FUNDING

This study/project is funded by the National Institute for Health Research (NIHR) School for Primary Care Research (project reference 365). J Sheppard receives funding from the Wellcome Trust/Royal Society via a Sir Henry Dale Fellowship (ref: 211182/Z/18/Z). H Ashdown has been funded by an NIHR Doctoral Research Fellowship (DRF-2014-07-052).

For peer review only - http://bmjopen.bmj.com/site/about/guidelines.xhtml

For peer review only

COMPETING INTERESTS

C Butler reports grants from the NIHR and grants from the NIHR Health Protection Research Unit during the conduct of the study; personal fees from Pfizer outside of the submitted work. J Sheppard reports grants from Wellcome Trust/Royal Society and from NIHR Oxford Biomedical Research Centre. H Ashdown has contributed to a COPD prescribing study using the CPRD and her academic department has received fees from Boehringer-Ingelheim for this work. C Bankhead reports grants from NIHR Oxford Biomedical Research Centre, grants from NIHR Applied Research Collaborative Oxford and Thames Valley during the conduct of the study.

AUTHOR CONTRIBUTIONS

M Smith planned and conducted the study, carried out statistical analyses and drafted the manuscript. H Ashdown provided clinical expertise on COPD. M Smith, H Ashdown, J Sheppard, C Butler, C Bankhead had full access to all the data in the study, contributed to the study design, interpretation of results, revisions of the manuscript, and approved the final manuscript.

ACKNOWLEDGEMENTS

C Bankhead and M Smith are supported by the National Institute for Health Research (NIHR) Oxford Biomedical Research Centre (BRC) and by the NIHR Applied Research Collaborative Oxford and Thames Valley. J Sheppard is supported by an NIHR Oxford BRC Senior Fellowship. The views expressed are those of the author(s) and not necessarily those of the NIHR or the Department of Health and Social Care. We thank the Patient and Public Involvement volunteers for their time and for sharing their knowledge.

ETHICS APPROVAL

CPRD has ethics approval from the Health Research Authority to support research using anonymised patient data. GP practices choose to share patient data (primary care data) with CPRD for public health research purposes. However, individual patients can opt out of sharing their data with CPRD. The project protocol was approved by the CPRD Independent Scientific Advisory Committee (protocol number 18_059A).

DATA SHARING STATEMENT

This study is based on data from the CPRD obtained under licence from the UK Medicines and Healthcare products Regulatory Agency. Information on how to access the data used for these analyses is available at https://www.cprd.com/home/. The work uses data provided by patients and collected by the NHS as part of their care and support and would not have been possible without

For peer review only - http://bmjopen.bmj.com/site/about/guidelines.xhtml

For peer review only

access to this data. The NIHR recognises and values the role of patient data, securely accessed and stored, in both underpinning and leading to improvements in research and care.

REFERENCES

1. Vogelmeier CF, Criner GJ, Martinez FJ, et al. Global Strategy for the Diagnosis, Management, and Prevention of Chronic Obstructive Lung Disease 2017 Report. GOLD Executive Summary. *American journal of respiratory and critical care medicine* 2017;195(5):557-82.
2. Divo M, Cote C, de Torres JP, et al. Comorbidities and risk of mortality in patients with chronic obstructive pulmonary disease. *American journal of respiratory and critical care medicine* 2012;186(2):155-61.
3. NICE. Cardiovascular disease: risk assessment and reduction, including lipid modification. *NICE guidelines [CG181]* Published date: July 2014 2014 Last updated: September 2016
4. Gan WQ, Man SFP, Senthilselvan A, et al. Association between chronic obstructive pulmonary disease and systemic inflammation: a systematic review and a meta-analysis. *Thorax* 2004;59(7):574-80.
5. Agusti A, Edwards LD, Rennard SI, et al. Persistent systemic inflammation is associated with poor clinical outcomes in COPD: a novel phenotype. *PloS one* 2012;7(5):e37483.
6. Quist-Paulsen P. Statins and inflammation: an update. *Current Opinion in Cardiology* 2010;25(4)
7. Cao C, Wu Y, Xu Z, et al. The effect of statins on chronic obstructive pulmonary disease exacerbation and mortality: a systematic review and meta-analysis of observational research. *Scientific reports* 2015;5:16461.
8. Ingebrigtsen TS, Marott JL, Nordestgaard BG, et al. Statin use and exacerbations in individuals with chronic obstructive pulmonary disease. *Thorax* 2015;70(1):33-40.
9. Walsh A, Perrem L, Khashan AS, et al. Statins versus placebo for people with chronic obstructive pulmonary disease. *The Cochrane database of systematic reviews* 2019;7(7):CD011959-CD59.
10. Suissa S. Immortal time bias in observational studies of drug effects. *Pharmacoepidemiology and drug safety* 2007;16(3):241-9.
11. Danaei G, Tavakkoli M, Hernan MA. Bias in observational studies of prevalent users: lessons for comparative effectiveness research from a meta-analysis of statins. *American journal of epidemiology* 2012;175(4):250-62.
12. Young RP, Hopkins RJ, Agusti A. Statins as adjunct therapy in COPD: how do we cope after STATCOPE? *Thorax* 2014;69(10):891-4.
13. Herrett E, Gallagher AM, Bhaskaran K, et al. Data Resource Profile: Clinical Practice Research Datalink (CPRD). *Int J Epidemiol* 2015;44(3):827-36.
14. Williams T, van Staa T, Puri S, et al. Recent advances in the utility and use of the General Practice Research Database as an example of a UK Primary Care Data resource. *Ther Adv Drug Saf* 2012;3(2):89-99.
15. Quint JK, Müllerova H, DiSantostefano RL, et al. Validation of chronic obstructive pulmonary disease recording in the Clinical Practice Research Datalink (CPRD-GOLD). 2014;4(7):e005540.
16. Danese MD, Gleeson M, Kutikova L, et al. Management of lipid-lowering therapy in patients with cardiovascular events in the UK: a retrospective cohort study. *BMJ open* 2017;7(5):e013851-e51.

For peer review only - http://bmjopen.bmj.com/site/about/guidelines.xhtml

17. Rothnie KJ, Mullerova H, Hurst JR, et al. Validation of the Recording of Acute Exacerbations of COPD in UK Primary Care Electronic Healthcare Records. *PloS one* 2016;11(3):e0151357.
18. Rothnie KJ, Mullerova H, Thomas SL, et al. Recording of hospitalizations for acute exacerbations of COPD in UK electronic health care records. *Clin Epidemiol* 2016;8:771-82.
19. Mullerova H, Shukla A, Hawkins A, et al. Risk factors for acute exacerbations of COPD in a primary care population: a retrospective observational cohort study. *BMJ open* 2014;4(12):e006171.
20. Calverley PM, Tetzlaff K, Dusser D, et al. Determinants of exacerbation risk in patients with COPD in the TIOSPIR study. *International journal of chronic obstructive pulmonary disease* 2017;12:3391-405.
21. Amorim LD, Cai J. Modelling recurrent events: a tutorial for analysis in epidemiology. *Int J Epidemiol* 2014;44(1):324-33.
22. Neukamm A, Hoiseth AD, Einvik G, et al. Rosuvastatin treatment in stable chronic obstructive pulmonary disease (RODEO): a randomized controlled trial. *Journal of internal medicine* 2015;278(1):59-67.
23. Criner GJ, Connett JE, Aaron SD, et al. Simvastatin for the prevention of exacerbations in moderate-to-severe COPD. *The New England journal of medicine* 2014;370(23):2201-10.
24. Schenk P, Spiel AO, Hüttinger F, et al. Can Simvastatin reduce COPD Exacerbations? A Randomised Double Blind Controlled Study. *The European respiratory journal* 2021
25. Morgan AD, Zakeri R, Quint JK. Defining the relationship between COPD and CVD: what are the implications for clinical practice? *Therapeutic advances in respiratory disease* 2018;12:1753465817750524.
26. Rabe KF, Hurst JR, Suissa S. Cardiovascular disease and COPD: dangerous liaisons? *European respiratory review : an official journal of the European Respiratory Society* 2018;27(149)
27. Collins R, Reith C, Emberson J, et al. Interpretation of the evidence for the efficacy and safety of statin therapy. *Lancet (London, England)* 2016;388(10059):2532-61.
28. He Y, Li X, Gasevic D, et al. Statins and Multiple Noncardiovascular Outcomes: Umbrella Review of Meta-analyses of Observational Studies and Randomized Controlled Trials. *Annals of internal medicine* 2018;169(8):543-53.
29. Chou R, Dana T, Blazina I, et al. Statins for Prevention of Cardiovascular Disease in Adults: Evidence Report and Systematic Review for the US Preventive Services Task Force. *Jama* 2016;316(19):2008-24.
30. Mansournia MA, Etminan M, Danaei G, et al. Handling time varying confounding in observational research. *BMJ (Clinical research ed)* 2017;359:j4587.
31. Ingebrigtsen TS, Marott JL, Nordestgaard BG, et al. Low use and adherence to maintenance medication in chronic obstructive pulmonary disease in the general population. *J Gen Intern Med* 2015;30(1):51-59.
32. Forde I, Chandola T, Raine R, et al. Socioeconomic and ethnic differences in use of lipid-lowering drugs after deregulation of simvastatin in the UK: the Whitehall II prospective cohort study. *Atherosclerosis* 2011;215(1):223-8.
33. Paudyal V, Hansford D, Cunningham S, et al. Pharmacists' perceived integration into practice of over-the-counter simvastatin five years post reclassification. *International journal of clinical pharmacy* 2012;34(5):733-8.

For peer review only - http://bmjopen.bmj.com/site/about/guidelines.xhtml

Figure 1. Selection of the study population

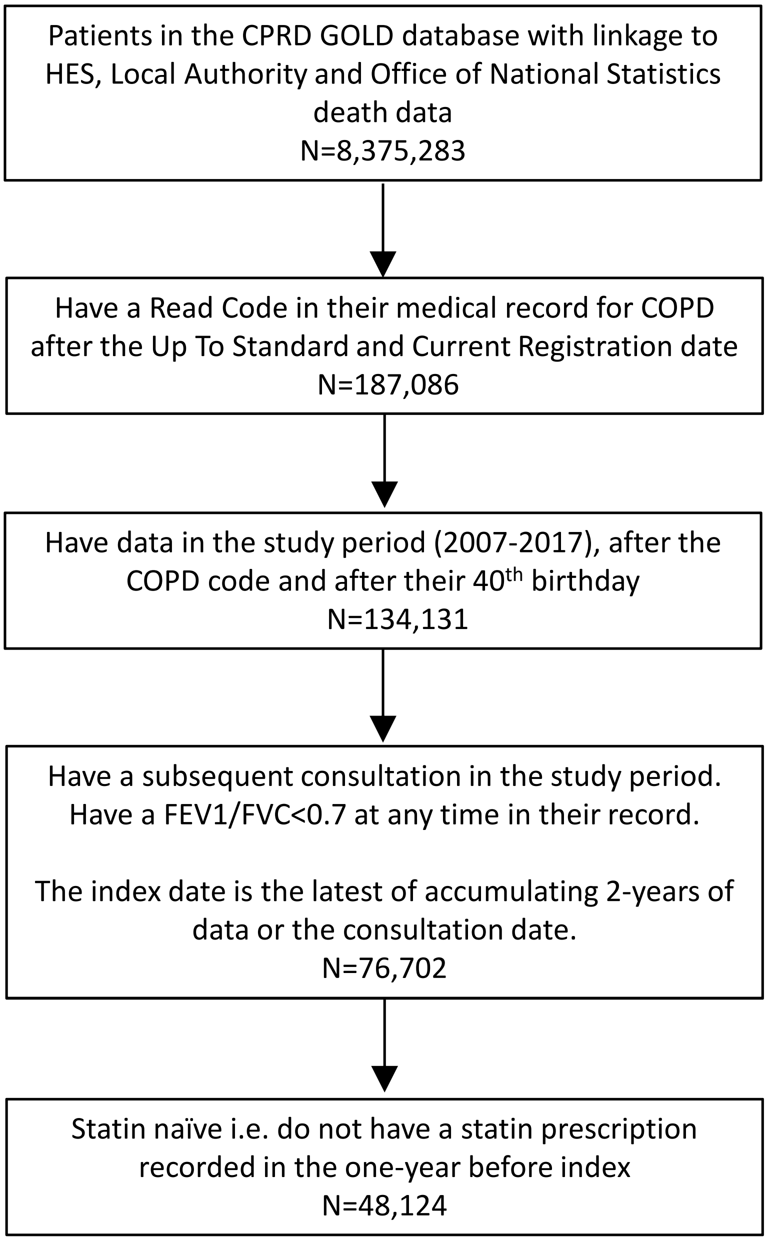

STROBE 2007 (v4) Statement—Checklist of items that should be included in reports of *cohort studies*

Section/Topic	Item #	Recommendation	Reported on page #
**Title and abstract**	1	(*a*) Indicate the study's design with a commonly used term in the title or the abstract	Title & Abstract
		(*b*) Provide in the abstract an informative and balanced summary of what was done and what was found	2-3
**Introduction**			
Background/rationale	2	Explain the scientific background and rationale for the investigation being reported	3-4
Objectives	3	State specific objectives, including any prespecified hypotheses	3
**Methods**			
Study design	4	Present key elements of study design early in the paper	4
Setting	5	Describe the setting, locations, and relevant dates, including periods of recruitment, exposure, follow-up, and data collection	4
Participants	6	(*a*) Give the eligibility criteria, and the sources and methods of selection of participants. Describe methods of follow-up	5
		(*b*) For matched studies, give matching criteria and number of exposed and unexposed	NA
Variables	7	Clearly define all outcomes, exposures, predictors, potential confounders, and effect modifiers. Give diagnostic criteria, if applicable	5
Data sources/ measurement	8*	For each variable of interest, give sources of data and details of methods of assessment (measurement). Describe comparability of assessment methods if there is more than one group	6
Bias	9	Describe any efforts to address potential sources of bias	4
Study size	10	Explain how the study size was arrived at	Fig 1
Quantitative variables	11	Explain how quantitative variables were handled in the analyses. If applicable, describe which groupings were chosen and why	7
Statistical methods	12	(*a*) Describe all statistical methods, including those used to control for confounding	7
		(*b*) Describe any methods used to examine subgroups and interactions	NA
		(*c*) Explain how missing data were addressed	7
		(*d*) If applicable, explain how loss to follow-up was addressed	5
		(*e*) Describe any sensitivity analyses	7
**Results**			

For peer review only

Participants	13*	(a) Report numbers of individuals at each stage of study—eg numbers potentially eligible, examined for eligibility, confirmed eligible, included in the study, completing follow-up, and analysed	Fig 1, p7
		(b) Give reasons for non-participation at each stage	Fig 1
		(c) Consider use of a flow diagram	Fig 1
Descriptive data	14*	(a) Give characteristics of study participants (eg demographic, clinical, social) and information on exposures and potential confounders	Table 1
		(b) Indicate number of participants with missing data for each variable of interest	Table 1
		(c) Summarise follow-up time (eg, average and total amount)	10
Outcome data	15*	Report numbers of outcome events or summary measures over time	10, Table 2
Main results	16	(*a*) Give unadjusted estimates and, if applicable, confounder-adjusted estimates and their precision (eg, 95% confidence interval). Make clear which confounders were adjusted for and why they were included	Table 2
		(*b*) Report category boundaries when continuous variables were categorized	P7, Table 2
		(*c*) If relevant, consider translating estimates of relative risk into absolute risk for a meaningful time period	NA
Other analyses	17	Report other analyses done—eg analyses of subgroups and interactions, and sensitivity analyses	Table2, Table 3
**Discussion**			
Key results	18	Summarise key results with reference to study objectives	P13
**Limitations**			
Interpretation	20	Give a cautious overall interpretation of results considering objectives, limitations, multiplicity of analyses, results from similar studies, and other relevant evidence	P13-p15
Generalisability	21	Discuss the generalisability (external validity) of the study results	P13-P15
**Other information**			
Funding	22	Give the source of funding and the role of the funders for the present study and, if applicable, for the original study on which the present article is based	P16

*Give information separately for cases and controls in case-control studies and, if applicable, for exposed and unexposed groups in cohort and cross-sectional studies.

Note: An Explanation and Elaboration article discusses each checklist item and gives methodological background and published examples of transparent reporting. The STROBE checklist is best used in conjunction with this article (freely available on the Web sites of PLoS Medicine at http://www.plosmedicine.org/, Annals of Internal Medicine at http://www.annals.org/, and Epidemiology at http://www.epidem.com/). Information on the STROBE Initiative is available at www.strobe-statement.org.

For peer review only
